# Apoptotic cell fragments locally activate tingible body macrophages in the germinal center

**DOI:** 10.1016/j.cell.2023.02.004

**Published:** 2023-03-02

**Authors:** Abigail K. Grootveld, Wunna Kyaw, Veera Panova, Angelica W.Y. Lau, Emily Ashwin, Guillaume Seuzaret, Rama Dhenni, Nayan Deger Bhattacharyya, Weng Hua Khoo, Maté Biro, Tanmay Mitra, Michael Meyer-Hermann, Patrick Bertolino, Masato Tanaka, David A. Hume, Peter I. Croucher, Robert Brink, Akira Nguyen, Oliver Bannard, Tri Giang Phan

**Affiliations:** 1 Garvan Institute of Medical Research, Darlinghurst, Sydney, NSW, Australia; 2 St Vincent’s Healthcare Clinical Campus, School of Clinical Medicine, Faculty of Medicine and Health, UNSW Sydney, Sydney, NSW, Australia; 3 MRC Human Immunology Unit, Nuffield Department of Medicine, MRC Weatherall Institute of Molecular Medicine, University of Oxford, Oxford, UK; 4 Department of Biology and Biochemistry, University of Bath, Bath, UK; 5 Département de Biologie, Université de Lyon, Lyon, France; 6 EMBL Australia, Single Molecule Science Node, School of Medical Sciences, UNSW Sydney, Sydney, NSW, Australia; 7 Department of Systems Biology and Braunschweig Integrated Center for Systems Biology (BRICS), Helmholtz Center for Infection Research, Rebenring 56, D-38106 Braunschweig, Germany; 8 Institute for Biochemistry, Biotechnology and Bioinformatics, Technische Universität Braunschweig, Braunschweig, Germany; 9 Centenary Institute and University of Sydney, AW Morrow Gastroenterology and Liver Centre, Royal Prince Alfred Hospital, Sydney, NSW, Australia; 10 Tokyo University of Pharmacy and Life Sciences, Tokyo, Japan; 11 Mater Research Institute, University of Queensland, Brisbane, QLD, Australia

## Abstract

Germinal centers (GCs) that form within lymphoid follicles during antibody responses are sites of massive cell death. Tingible body macrophages (TBMs) are tasked with apoptotic cell clearance to prevent secondary necrosis and autoimmune activation by intracellular self antigens. We show by multiple redundant and complementary methods that TBMs derive from a lymph node-resident, CD169-lineage, CSF1R-blockade-resistant precursor that is prepositioned in the follicle. Non-migratory TBMs use cytoplasmic processes to chase and capture migrating dead cell fragments using a “lazy” search strategy. Follicular macrophages activated by the presence of nearby apoptotic cells can mature into TBMs in the absence of GCs. Single-cell transcriptomics identified a TBM cell cluster in immunized lymph nodes which upregulated genes involved in apoptotic cell clearance. Thus, apoptotic B cells in early GCs trigger activation and maturation of follicular macrophages into classical TBMs to clear apoptotic debris and prevent antibody-mediated autoimmune diseases.

## Graphical abstract

**Graphical abstract:**
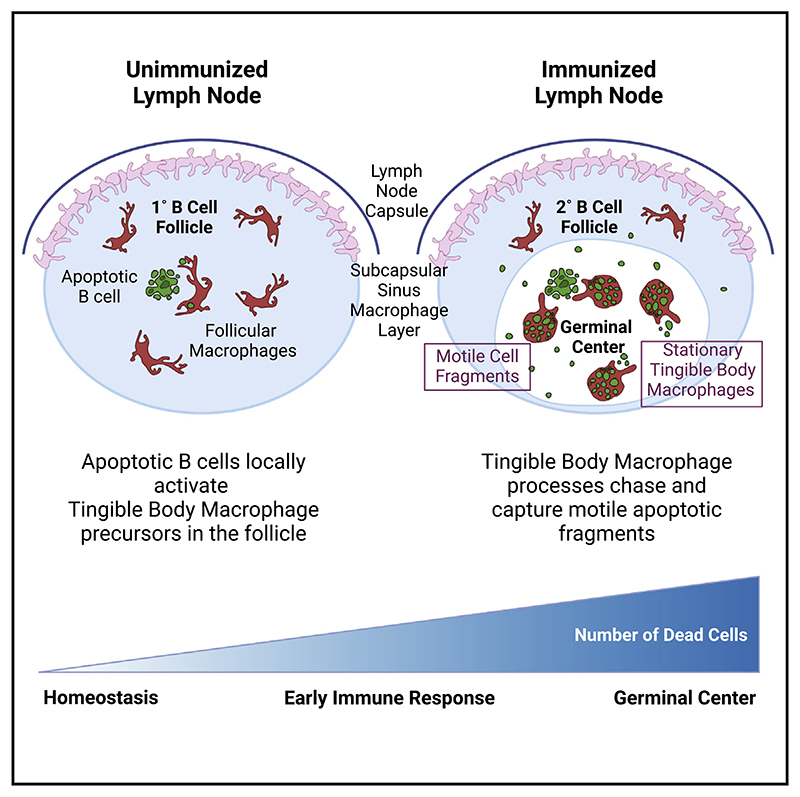

## Introduction

Germinal centers (GCs) are transient structures that develop in lymph nodes and the spleen in response to infection and vaccination.^1^ B cells activated by antigens are recruited to GCs where they hypermutate their B cell receptor (BCR) and compete for T cell help in the light zone to refuel their continued proliferation in the dark zone.^2^ B cells that acquire damaging mutations or fail to receive T cell help,^3,4^ and those that acquire self reactivity,^5^ undergo apoptotic cell death. Accordingly, the GC is a site of both intense cellular proliferation, where activated B cells divide at least 2–4 times a day,^6–8^ and intense cell death, where up to half of the GC B cells are estimated to die every 5.3 h.^3^ The efficient clearance of the cytoplasmic and nuclear debris generated by this extensive cell death is a critical housekeeping function of professional scavengers in the GC called tingible body macrophages (TBMs).^9–11^

During apoptosis, the exposure of phosphatidylserine on the outer leaflet of the plasma membrane signals “eat me” to macrophages.^12^ In the GC, milk fat globule epidermal growth factor-8 (MFGE8), secreted by follicular dendritic cells (FDCs), acts as a molecular bridge by binding phosphatidylserine on apoptotic cells and integrins on TBMs via its RGD motif.^13^ Mice deficient for MFGE8 have impaired engulfment of apoptotic cells by TBMs, expanded GCs, anti-nuclear autoantibodies, and immune complex-mediated glomerulonephritis.^14^ TBMs express the Mer tyrosine kinase (MERTK) phagocytic receptor^15^ and MERTK-deficient mice similarly have impaired apoptotic cell clearance, expanded GCs, autoantibody production, and lupus-like autoimmunity.^16,17^ Thus, TBMs prevent the extrafollicular spread of self antigens, and defective apoptotic cell clearance has been implicated in the pathogenesis of systemic lupus erythematosus (SLE).^18–22^

Most of our knowledge about TBMs derives from static analyses of immunohistochemistry performed on tissue sections. Classical TBMs containing the ingested corpses of dead cells (“tingible bodies”) have not been reported in the resting lymph node and only become evident once GCs have formed after vaccination or infection.^23^ However, it is unclear if TBM-like cells or their precursors are present in a resting state in the primary B cell follicle. The floor of the subcapsular sinus (SCS) in the lymph node is lined by CD169^+^ macrophages that sample the lymph for antigens, forming an interconnected network overlying the B cell follicle.^24,25^ Infection and inflammation are associated with disruption of this CD169^+^ SCS macrophage (SSM) cell layer,^26–28^ possibly due to their inward displacement into the follicle.^26^ It has therefore been proposed that SSMs that migrate into the follicle may also differentiate into TBMs.

Here, we use a CD169-lineage reporter mouse^29^ to test the SSM hypothesis. While this mouse did mark TBMs, the cells themselves did not express cell-surface CD169 and were able to develop even when SSMs were depleted by colony-stimulating factor-1 receptor (CSF1R) blockade. Intravital imaging revealed that TBMs were stationary and used cellular processes to chase and capture apoptotic cell fragments. Photoconversion, lead-shielded irradiation, and additional lineage tracing established that TBMs originated from a long-lived lymph node-resident cell population. Single-cell RNA sequencing (scRNA-seq) revealed a population of putative TBMs which were activated to upregulate genes involved in apoptotic cell capture and clearance in immunized lymph nodes. Analysis of resting lymph nodes confirmed the presence of macrophages, but with few tingible bodies, in the primary follicle. We show by inducible B cell killing and localized intravital two-photon photoablation that these macrophages can locally engulf apoptotic B cells and acquire mature TBM characteristics, even in the absence of a GC. Thus, macrophages residing in the follicle can mature in the presence of apoptotic cells to become classical TBMs.

## Results

### TBMs are derived from CD169-lineage cells

**Figure 1:**
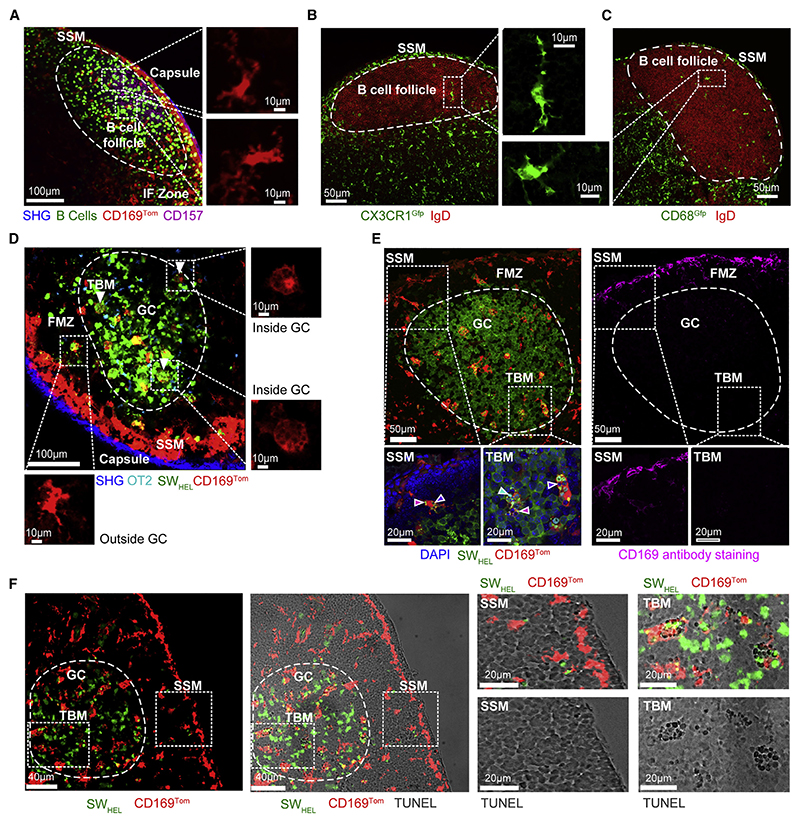
Visualization of TBMs by CD169, CX3CR1, and CD68 reporters (A) Maximum intensity projection (MIP) of the inguinal lymph node of an unimmunized CD169^Tom^ mouse. SHG, second harmonic generation (blue); green, naive Kaede B cells; red, tdTomato; magenta, follicular stroma labeled with anti-CD157 antibody. SSM; subcapsular sinus macrophage; IF, interfollicular zone. (B and C) Confocal images of inguinal lymph nodes of unimmunized CX3CR1^Gfp^ (B) and CD68^Gfp^ mice (C) stained for IgD (red). (D) MIP of CD169^Tom^ inguinal lymph node 10 days after HEL-OVA immunization. SHG, second harmonic generation (blue); cyan, CFP OT-II T cells; green, Kaede SW_HEL_ GC B cells. SSM; subcapsular sinus macrophage; FMZ, follicular mantle zone; GC, germinal center; TBM, tingible body macrophage. *See also* Video S1. (E) Confocal image of day 8 Kaede SW_HEL_ GC in a CD169^Tom^ mouse. Sections were stained with CD169 (magenta) and DAPI (blue). Arrowheads indicate vacuoles containing DAPI only (purple); Kaede only (pink); DAPI and Kaede (cyan). (F) Combined fluorescent and brightfield image day 8 Kaede SW_HEL_ GC in a CD169^Tom^ mouse showing TUNEL staining.

To image CD169-lineage cells, we generated *Cd169^Cre/+^. LSL-Tdtomato* (CD169^Tom^) mice in which cells that had turned on the *Si-glec1* gene and their progeny were irreversibly marked with the red-fluorescent protein tdTomato (TOM). This reporter labels several macrophage and dendritic cell populations in the floor of the SCS, medullary sinus, interfollicular zone, B cell follicle, and T cell zone of unimmunized lymph nodes (Figure S1A). Intravital mi-croscopy revealed that TOM+ macrophages inside the follicle had multiple dendritic cellular processes (Figure 1A). Similar macrophages with dendritic morphology were also observed in the follicles of CX3CR1^Gfp^ (Figure 1B) and CD68^Gfp^ mice (Figure 1C). We next established GCs containing green-fluorescent Kaede SW_HEL_ B cells and cyan OT-II T cells in recipient CD169^Tom^ mice to track TBM dynamics in response to immunization with henegg lysozyme (HEL) conjugated to ovalbumin (OVA).^30,31^ Intravital microscopy of immunized lymph nodes on day 10 revealed larger, rounder, more vacuolated TOM+ macrophages inside the GC, in addition to dendritic TOM+ macrophages in the follicular mantle zone (FMZ) outside the GC (Figure 1D; Video S1). Confocal microscopy revealed that TOM+ cells in GCs of CD169^Tom^ mice did not stain with antibodies to CD169, whereas the macrophages in the FMZ had low levels of staining (Figure 1E). The presence of TUNEL-positive apoptotic bodies inside the vacuoles confirmed that the cells were TBMs (Figure 1F). Confocal analysis showed that red GC macrophages had a more rounded morphology and 85% (95% CI: 75%–94%) contained multiple green-fluorescent B cell fragments inside vacuoles. Moreover, 87% (95% CI: 81%–94%) of TBMs contained TUNEL+ nuclear debris inside vacuoles. GC TBMs also expressed GFP in CD68^Gfp^ and CX3CR1^Gfp^ mice (Figures S1B and S1C). FACS showed that in immunized CD169^Tom^ mice, 70% of TOM^+^MERTK^+^CD169^–^CD11b^+^ cells expressed CX3CR1 (Figure S1D). Confocal analysis of TOM+ cells inside the GC revealed the subcellular localization of the late endosome marker CD68 inside phagocytic vacuoles (Figure S1E). TBMs were evident in CX3CR1-deficient mice, indicating that they were CX3CR1 independent (FigureS1F). Thus, the resting follicle harbored dendritic CD169-lineage macrophages distinct from SSMs, and the immunized follicle contained additional rounded CD169-lineage macrophages that exhibited more classical features of TBMs.

### Cellular dynamics of TBMs in the GC

**Figure 2:**
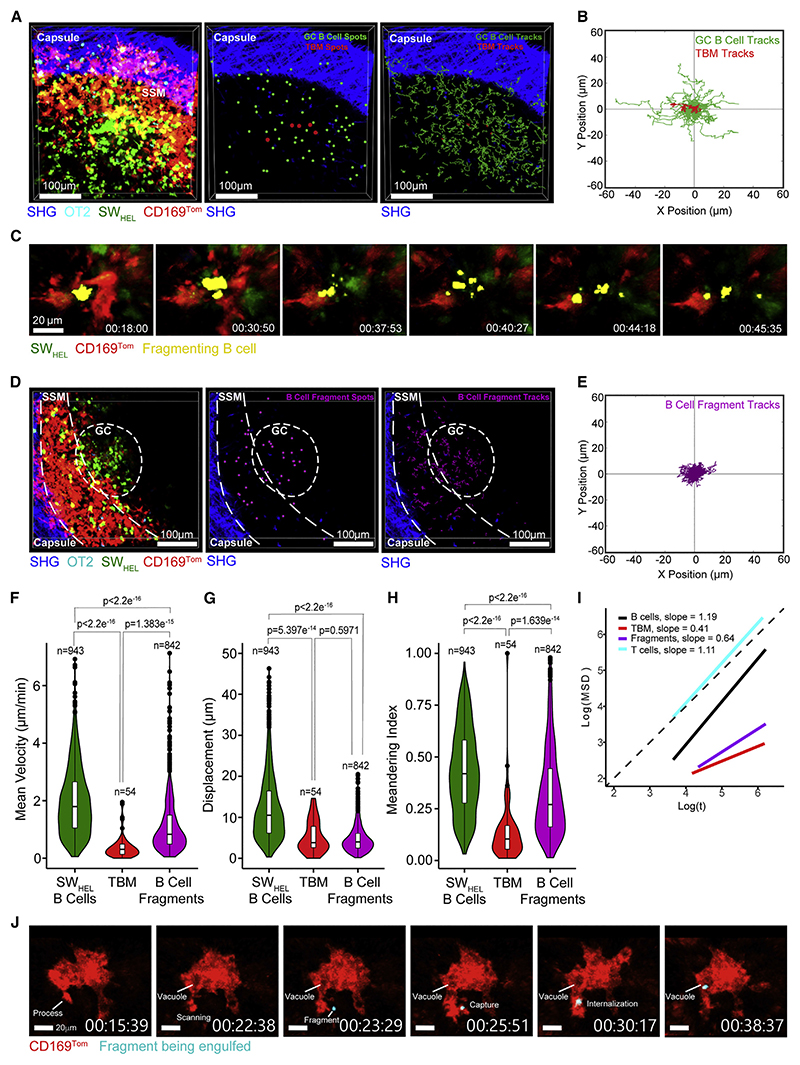
Stationary TBMs use processes to chase and capture motile dead cell fragments (A) MIP of day 10 GC in CD169^Tom^ mice showing the imaging volume (left panel), spot detection (middle panel), and cell tracks (right panel). SHG (blue); OT-II T cells (cyan); Kaede B cells (green). *See also* Video S2. (B) Tracks of GC B cells (green) and TBMs (red) centered on the same origin. (C) Time-lapse images from day 14 GC in a CD169^Tom^ lymph node showing Kaede SW_HEL_ B cells (green) fragmenting (pseudocolored yellow) next to a TBM (red). *See also* Video S3. (D) MIP of day 10 GC in CD169^Tom^ mice showing the imaging volume (left panel), spot detection of B cell fragments (middle panel), and cell fragment tracks (right panel). SHG (blue); OT-II T cells (cyan); Kaede B cells (green). *See also* Video S4. (E) Tracks of apoptotic cell fragments (magenta) centered on the same origin. (F) Violin plot of mean velocity for GC B cells (green, n = 943), TBMs (red, n = 54), and GC B cell fragments (pink, n = 842). n represents individual cells/fragments and data are representative of at least 3 independent movies. (G) Violin plot of displacement for GC B cells (green, n = 943), TBMs (red, n = 54), and GC B cell fragments (pink, n = 842). n represents individual cells/fragments and data are representative of at least 3 independent movies. (H) Violin plot of meandering index for GC B cells (green, n = 943), TBMs (red, n = 54), and GC B cell fragments (pink, n = 842). n represents individual cells/ fragments and data are representative of at least 3 independent movies. (I) Mean squared displacement (MSD) plot of apoptotic fragments, GC B cells, GC T cells, and TBMs. (J) Time-lapse images from day 14 GC in a CD169^Tom^ lymph node showing a TBM (red) extending a process to capture an apoptotic cell fragment (pseudocolored cyan). *See also* Video S5.

Intravital imaging revealed that, in contrast to motile GC B cells, TBMs were stationary and did not migrate in search of apoptotic cells (Figures 2A and 2B; Video S2). Apoptotic GC B cells were observed to fragment, often in the vicinity of TBMs (Figure 2C; Video S3). Tracking of apoptotic cell fragments based on their size (Figure S1G) showed that they were motile and able to migrate within the GC (Figures 2D and 2E; Video S4). Some fragments were located outside the GC in the subcapsular region (Figure 2D). Analysis of motility parameters confirmed that TBMs were non-migratory with slower mean velocity (Figure 2F) and displacement (Figure 2G) than GC B cells. In contrast, GC B cell fragments were more migratory than TBMs but less motile than B cells (Figure 2H). Unlike GC B cells and B cell fragments, TBMs were locally confined with a meandering index <0.25 (Figure 2H). GC B and T cells were superdiffusive with slope a α1, and TBMs and fragments were subdiffusive with a α 1 (Figure 2I). TBMs were nevertheless dynamic and extended multiple cellular processes that actively sampled the local environment and could be observed chasing and capturing apoptotic cell fragments (Figure 2J; Video S5).

### TBMs adopt a “lazy” but efficient search strategy

**Figure 3:**
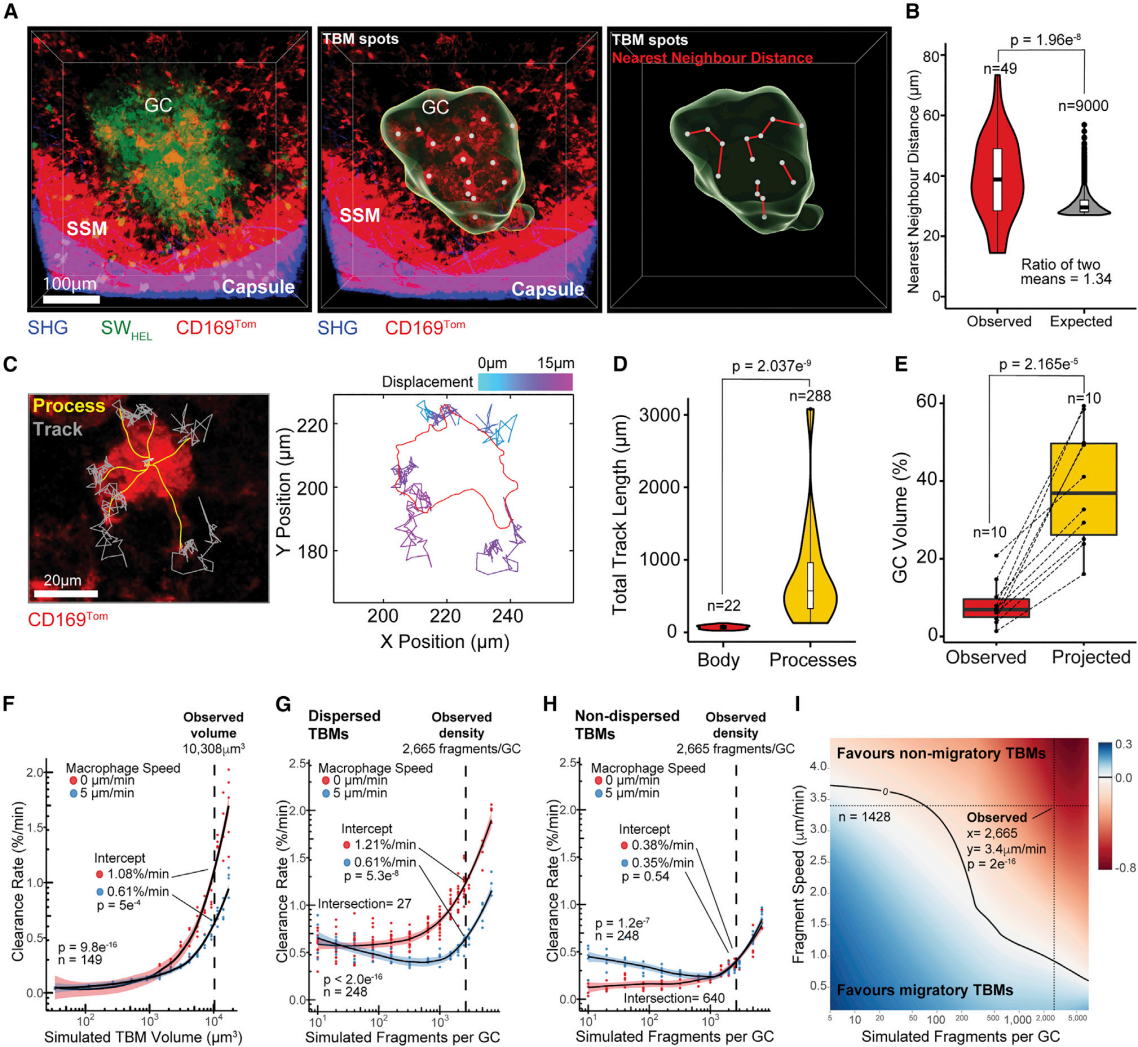
TBMs use a “lazy” search strategy to capture apoptotic cell fragments (A) MIP of day 14 GC in CD169^Tom^ mice showing the imaging volume (left panel), spot detection of TBMs and GC surface (middle panel), and nearest neighbor distance between TBM centroids (right panel). SHG (blue); Kaede B cells (green). (B) Nearest neighbor distances between observed TBMs (red) in the GC and Monte Carlo sampling of randomly generated points in an equivalent simulated sphere of radius 81μm. (C) Analysis of TBM imaged in Figure 2J by filament tracing (left panel) the cell processes with filaments (yellow) and the tracks of the endpoints of the filaments (gray). TBM contour (right panel) shows the outline of the TBM (red) and the cell processes pseudocolored for their total track displacement. (D) Comparison of the total track displacement of TBM centroids (red) to cell processes (yellow). n = number of TBMs or cell processes tracked. (E) Time projection showing increase in the effective TBM volume from observed (red) to projected (yellow) as a proportion of total GC volume for n = 10 GCs. (F) Simulated TBM volume on clearance rate for non-migratory (red) and migratory (blue) TBMs moving at a speed of 5 μm/min (n = 149 simulations). (G) Simulated effect of fragment abundance on clearance rate for non-migratory (red) and migratory (blue) TBMs moving at a speed of 5 μm/min (n = 248 simulations) in GC with dispersed TBMs. (H) As (G) in GC with non-dispersed randomly distributed TBMs (n = 248 simulations). (I) Optimal search conditions for apoptotic cell clearance. Differences in clearance rates for migratory and non-migratory TBMs were simulated for cell fragment speed and abundance (n = 1,428 simulations). Negative difference in clearance rates (red area) indicate conditions which favor more rapid clearance by stationary macrophages; positive differences (blue area) indicate conditions which favor more rapid clearance by migratory macrophages. The observed conditions are indicated by the dashed lines. Horizontal dashed line = cell fragment speed of 3.4 μm/min. Vertical dashed line = density of 2,665 cell fragments per GC.

Typically, “predator” cells migrate in search of their “prey.”^31,32^ In contrast, non-migratory TBMs scanned for apoptotic cell fragments using cellular processes in what appeared to be an unusually “lazy” search strategy. We performed mathematical modeling to analyze this behavior (STAR*Methods; Figures S3A–S3E). Each GC volume of 2.23 × 10^6^μm^3^ contained 18 TBMs (95% CI: 10–27) that were evenly dispersed and positioned with in 41μm of their nearest neighbor (Figures 3A and 3B). Monte Carlo simulations of the same number of TBMs randomly distributed within the same GC volume showed that the nearest neighbor distance in this scenario was significantly shorter at 30.5μm. The nearest neighbor index (ratio of observed to expected) of 1.34 (p = 5.4 × 10^–8^) indicated that TBMs are dispersed non-randomly (Figure 3B). While the TBM cell bodies did not move, the cell processes were highly motile, and analysis of their displacement showed that they were anchored and reset to the same parts of the cell (Figure 3C). The cumulative track lengths of the processes covered distances that were exponentially longer than those covered by the stationary cell bodies (Figure 3D). Time projection showed that the cell processes allowed TBMs to survey a volume that was 6.12-fold larger (p = 2.165 × 10^–5^) than the cell body (Figure 3E).

Agent-based modeling showed that the clearance rate of lazy stationary TBMs increased at a faster rate than that of migratory TBMs as the cell volume increased (p = 9.76 × 10^–16^)(Figure 3F). At the observed TBM volume of 10,308μm^3^, the clearance rate for migratory TBMs was 0.61%/min (95% CI: 0.58–0.63) compared to 1.08%/min (95% CI: 1.02–1.14) for stationary TBMs (p = 0.0005). We next simulated the removal of migrating apoptotic cell fragments by TBMs that were uniformly dispersed, and this showed that when prey is abundant, the clearance rate is higher for stationary than migratory TBMs (p < 2 × 10^–16^) (Figure 3G). At the observed density of 2,665 fragments per GC, the clearance rate was 1.21%/min (95% CI: 1.16–1.26) for non-migrating TBMs compared to 0.61%/min (95% CI: 0.56–0.66) for migrating TBMs (p = 5.3 × 10^–8^). We next simulated the removal of migrating apoptotic cell fragments by TBMs that were randomly distributed and non-dispersed (Figure 3H). Under these circumstances, the overall clearance rate was slower than for the dispersed TBMs (Figure 3G) and favored migratory TBMs at densities below 640 fragments per GC (95% CI: 416–1,230; p = 1.2 × 10^–7^). However, at the observed fragment density of 2,665 per GC, there was no difference between the clearance rate of 0.38%/min (95% CI: 0.34–0.41) for migrating TMBs compared to 0.35%/min (95% CI: 0.31–0.39) for non-migrating TBMs (p = 0.54). Thus, TBM migration is only advantageous at low prey densities. A broad parameter search showed that the optimal search strategy for TBMs was dependent on the density and motility of the fragments and favored stationary TBMs under observed conditions (p = 2 × 10^–16^) (Figure 3I).

### Single-cell RNA sequencing of CD169-lineage cells

**Figure 4:**
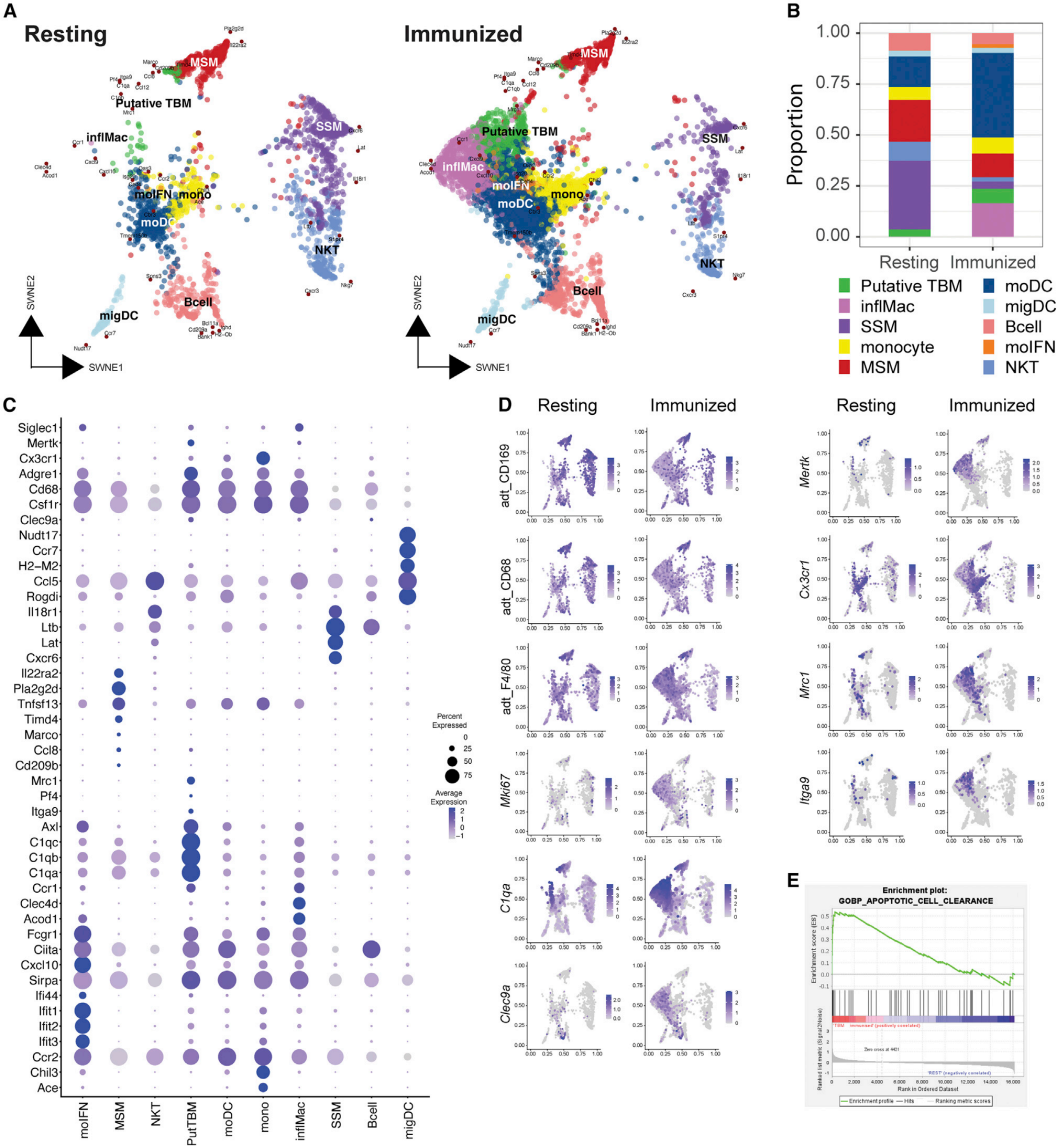
Single cell transcriptomic analysis of CD169-lineage cells (A) SWNE plot of NMF-decomposed single-cell gene expression data. Genes are embedded in the SWNE plot with respect to their relative contribution to each cluster. (B) Stacked bar plot showing the proportion of each cell population in the resting and immunized state. (C) Mean expression of driver genes defining each cluster and key genes involved in macrophage biology. The size of the dots relate to the proportion of cells in which expression is detected. (D) CITE-seq expression of cell surface proteins and genes of interest. (E) Enrichment plots from GSEA comparing immunized putative TBM to all other populations against gene ontology (GO) genesets.

We next sorted live CD11b^+^TOM^+^ cells by FACS and performed scRNA-seq by simultaneous epitope and transcriptome mapping (CITE-seq) of 11,384 single cells (Figures 4A and S2A). We decomposed the gene expression matrix by non-negative matrix factorization (NMF)^33^ to show that the data could be modeled by different factorization matrices with cophenetic correlation coefficients approaching 1.00 (Figure S2B). We selected the model with rank *k* = 10 clusters, as it provided the best solution in terms of accurately capturing the complexity of the data (cophenetic coefficient of 0.97) and revealing the subclusters that match the heterogeneity observed in the imaging. We embedded the cells and genes in a 2D visualization using similarity-weighted non-negative embedding (SWNE)^34^ to identify dynamic changes in cell clusters and gene expression from the resting to immunized state (Figure 4A). Cell populations were manually classified as SSMs, MSMs, monocytes, monocyte-derived DCs, migratory DCs, interferon-activated monocytes, inflammatory macrophages, and putative TBMs, as well as contaminating B cells and NKT cells (Figure 4B), based on the differential expression of canonical and non-canonical genes and cell-surface markers (Figures 4C and 4D). Cells in the putative TBM cluster express *Mertk*, *Cd68*, and *Cx3cr1*, and downregulate *Siglec1* mRNA and CD169 cell surface protein (Figures 4C and 4D), consistent with the imaging data and FACS analysis (Figures 1A–1E, S1D, and S1E). Putative TBMs had detectable *Adgre1* (encoding F4/80), *Axl*, *C1qa, C1qb* and *C1qc* (Figure 4C). F4/80 protein expression was confirmed by the detection of CITE-seq antibody staining (Figure 4D) and FACS analysis (Figure S1D). In published immunohistochemistry studies, F4/80 was detected on MSMs^35^ but was undetectable on TBMs.^16,36^ However, it has been reported to be expressed by 50% of TBMs by light and electron microscopy.^37^ A subset of putative TBMs and cells that clustered with MSMs expressed *Mrc1* encoding the mannose receptor CD206 (Figures 4C and 4D). FACS revealed CD206 staining on a subset of TOM^+^CD11b^+^CD169^-^ cells, which were MERTK^+^ and CX3CR1^+^, in addition to a population of TOM^+^CD11b^+^CD169^lo^ cells (Figure S2C). Confocal microscopy revealed CD206 staining of a subset TBMs and MSMs (Figure S2D). Putative TBMs expressed *Mki67* at low levels (Figure 4D). CX3CR1-CreERT2 was used to express a histone mCherry specifically in the nuclei of TBMs and distinguish them from the nuclei/DNA of engulfed apoptotic cells. While we could not detect expression of nuclear Ki67 protein, we showed that TBMs stained for EdU, consistent with recent cell division (Figure S2E). Putative TBMs expressed *Csf1r* (Figure 4C) and were previously shown to express a *Csf1r*-EGFP reporter transgene,^38^ but lacked detectable CSF1R protein.^39^ Gene set enrichment analysis (GSEA) showed that, compared to the other CD11b^+^TOM^+^ cell populations, putative TBMs from immunized mice were enriched for genes involved in apoptotic cell clearance, chemokine receptor binding, response to chemokine, and walking behavior. (Figure 4E; Tables S1 and S2). These data suggest that putative TBMs contains transcriptionally distinct CD169-lineage cells that are adapted to search for and remove apoptotic cell debris.

### TBMs are lymph node-resident macrophages

**Figure 5:**
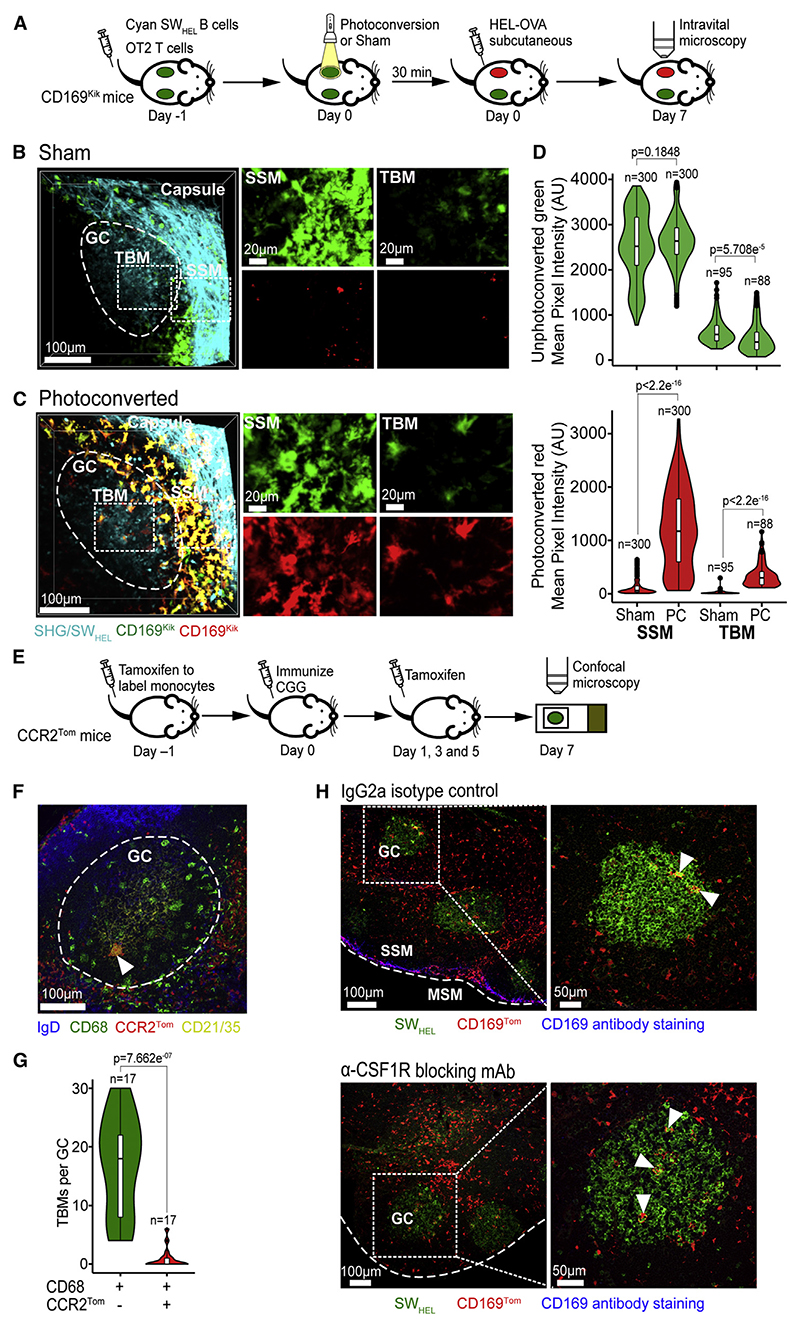
TBMs are derived from lymph node-resident macrophages (A) Schematic of photoconversion experiment to optically highlight CD169-lineage cells in the lymph nodes of CD169^Kik^ mice prior to immunization. (B) MIP of sham photoconverted control lymph node. (C) MIP of photoconverted lymph node. (D) Violin plot showing unphotoconverted green signal mean pixel intensity (top panel) and photoconverted red signal mean pixel intensity (bottom panel) for SSMs and TBMs in sham and photoconverted lymph nodes. n = number of individual cells; data are representative of 3 independent experiments. (E) Schematic for fate mapping the contribution of circulating monocytes to TBM pool using CCR2^Tom^ mice adoptively transferred into wildtype mice immunized with CGG in ASO3-like adjuvant. (F) MIP confocal image of day 7 GC showing follicular mantle (IgD, blue), follicular dendritic cell network (CD21/CD35, mustard), macrophages (CD68, green), and monocyte-derived cells (tdTomato, red). A single monocyte-derived TBM (arrow) is shown. (G) Quantification of (F) from multiple mice/GCs. Number and percentage of CD68^+^ TBMs in the GC that are CCR2^Tom^ positive or negative (n = number of GCs). Data from six mice and two independent experiments. (H) Confocal image of day 8 lymph nodes of CD169^Tom^ mice treated with IgG2a isotype control antibody (left panel) or anti-CSF1R blocking antibody (right panel). Depletion of SSMs and MSMs are shown by CD169 antibody staining (blue). Kaede SW_HEL_ GC B cells is shown in green.

To track the source of TBMs, we generated *Cd169^Cre/+^.LSL-Kikume* (CD169^Kik^) mice carrying the green-to-red photoconvertible fluorescent protein Kikume^40^ (Figure 5A). The inguinal lymph nodes in CD169^Kik^ mice were photoconverted with white light^41^ to irreversibly stamp all CD169-lineage cells at the time as red (Figure S4A). Mice were then immunized and TBMs analyzed on day 7 for expression of green and red fluorescence signals (Figures 5B–5D). In the follicle, SSMs were photoconverted, consistent with their known tissue residence. The majority of TBMs (98%) were also photoconverted (Figures 5C and S4B). A tissue-resident origin was also suggested by experiments in which individual lymph nodes in CD68^Gfp^ mice were shielded with lead during lethal whole-body irradiation >8 weeks prior to immunization and reconstituted with wildtype bone marrow (Figures S4C and S4D). Fate mapping of CX3CR1-expressing cells revealed that naive follicle macrophages were stably labeled and that TBMs developed from precursors expressing CX3CR1 three weeks prior to immunization (Figures S4E–S4G). To explore the contribution of non-tissue-resident circulating monocytes, *Ccr2^CreER/+^.Rosa^LSL-Tdtomato/+^*(CCR2^Tom^) mice were treated with tamoxifen just before and throughout the immune response to indelibly label CCR2^+^ inflammatory monocytes with red-fluorescent protein (Figures 5E and S4H). This confirmed that, while the majority of TBMs (>95%) are derived from non-CCR2-expressing tissue-resident cells, CCR2-expressing cells can make minor contributions to the TBM pool (Figures 5F and 5G). Bone marrow-derived cells were capable of fully reconstituting TBM populations when all tissue-resident precursors were ablated with lethal irradiation (Figures S4I and S4J).

Tissue-resident macrophages are typically dependent on CSF1R signaling for their development,^42^ and CSF1R blockade or *Csf1* mutation has been shown to deplete CD169^+^ SSMs, medullary sinus macrophages (MSMs), and tissue-resident monocytes.^43,44^ We therefore blocked CSF1R prior to and during the GC response in CD169^Tom^ mice (Figure 5H and Figure S4K). This efficiently depleted SSMs and MSMs in resting and immunized mice (Figure S4K and S4L). However, despite their expression of the *Csf1r* gene (Figure 4B), CSF1R blockade did not impair the generation of TBMs (Figures 5H and S4M). These data show that TBMs derive from a CSF1R-blockade-resistant lymph node-resident macrophage that is distinct from CSF1R-blockade-sensitive SSMs and MSMs.

### Distinct TBM morphologies in primary follicles and GCs

**Figure 6:**
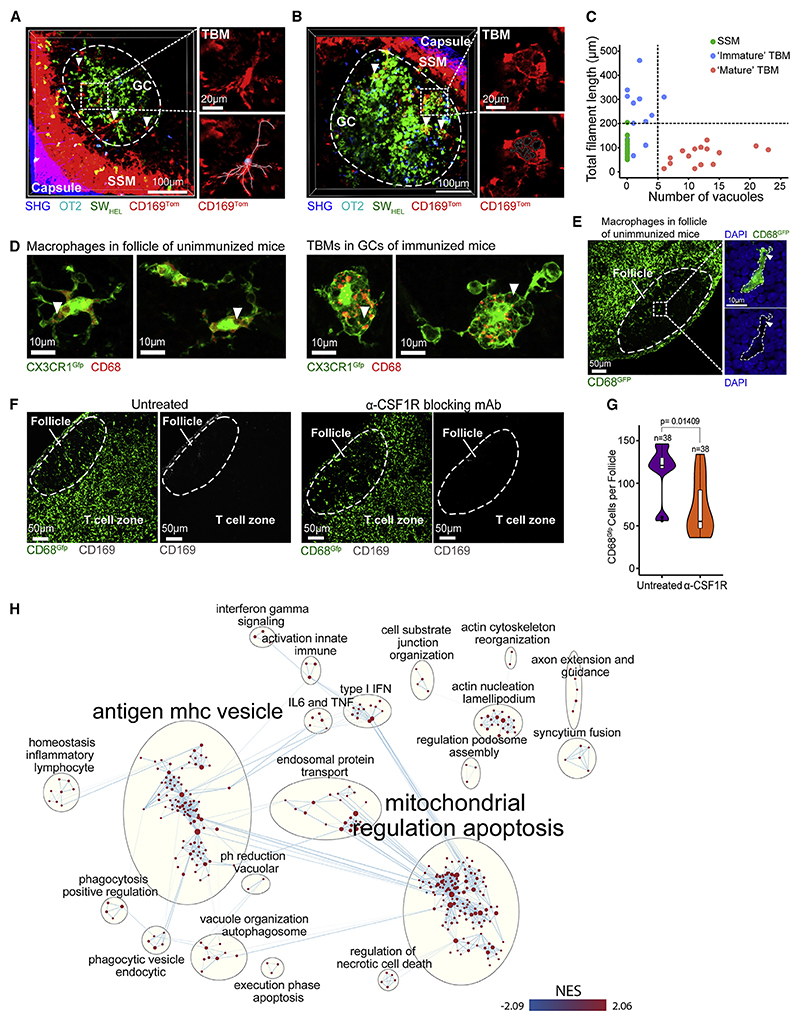
TBMs adopt distinct morphologies depending on their maturation state (A) MIP of day 9 GC in CD169^Tom^ mice showing an immature TBM inside the GC with long cytoplasmic processes and few vacuoles. (B) MIP of day 10 GC in CD169^Tom^ mice showing a mature, rounded TBM inside the GC with short cytoplasmic processes and multiple vacuoles. (C) Dot plot showing total measured filament length versus number of vacuoles for distinguishes 3 populations of TOM + macrophages: immature TBMs (blue), mature TBMs (red) and SSMs (green). (D) MIP confocal images of macrophages containing vacuoles (arrows) in naive follicles (unimmunized, left panels) and GCs (s.c. immunized CGG in ASO3-like adjuvant day 7, right panels) from inguinal lymph nodes of CX3CR1^Gfp^ mice. Antibody staining for CD68 (red). (E) MIP confocal images of lymph nodes from unimmunized CD68^Gfp^ mice stained with DAPI (blue). (F) MIP confocal images of lymph nodes from control and anti-CSFR1-treated CD68^Gfp^ unimmunized mice. B cell follicles were demarcated using IgD (not shown). CD169 antibody staining marks SSMs (white). (G) Quantitation of (E), showing number of CD68-GFP+ macrophages per follicle. N = number of follicles examined. Data compiled from 4 mice and 2 independent experiments. (H) Network of GSEA pathways enriched in immunized TBM compared to resting TBM. Each node represents a GO pathway, with the color representing the normalized enrichment score (NES).

The intravital imaging suggested that there were two macro-phage populations inside the follicle, based on their morphology (dendritic versus vacuolated) and location (FMZ versus GC) in immunized mice (Figure 1D). We reviewed GCs at different stages of maturation and observed similar differences in the morphology of GC TBMs (Figures 6A and 6B). Quantitative analysis of the intravital imaging distinguished three populations of TOM+ cells in the follicle, based on cell process length and number of phagocytic vacuoles (Figure 6C). SSMs had shorter processes and no vacuoles; immature TBMs had longer processes and occasionally contained vacuoles; and mature TBMs had shorter processes and multiple vacuoles.

The scRNA-seq data suggested that putative TBMs were present in unimmunized lymph nodes. We compared the morphology of GFP+ macrophages in the follicle of unimmunized to immunized CX3CR1^Gfp^ mice and showed that in the absence of GCs, some of these cells also contained the occasional CD68^+^ phagocytic vacuoles, similar to immature TBMs (Figure 6D). These vacuoles also contained DAPI+ DNA (Figure 6E). However, these vacuoles were not as prominent or numerous as those in GC TBMs. While blocking CSF1R in unimmunized CD68^Gfp^ mice depleted SSMs, follicular macrophages were still present, albeit at a reduced level (Figures 6F and 6G). Some of these GFP^+^ macrophages were peripherally located beneath the SCS but were distinct from SSMs, which are CD68 negative and CSF1R dependent. Thus, the majority of macrophages in the follicles of unimmunized mice were a CSF1R-blockade-resistant population that had TBM-like properties.

We next compared the transcriptome of the putative TBM cluster in unimmunized and immunized mice (Figure 6H). GSEA showed that immunization activates a number of key cellular processes (Figure 6H; Tables S3 and S4). In addition to genes related to innate immunity, IL-6 and TNF, and type I and type II interferon signaling pathways, immunization upregulated a number of processes related to apoptosis (such as mitochondrial regulation apoptosis, phagocytosis positive regulation, phagocytic vesicle endocytic, vacuole organization and autophagosome, and antigen mhc vesicle) and cell motility (such as actin cytoskeleton rearrangements, actin nucleation lamellipodium, axon guidance and extension, and regulation podosome assembly). Thus, cells classified as putative TBMs in resting lymph nodes upregulate genes to search for and clear apoptotic cells upon activation and maturation in immunized lymph nodes.

### Dead cells locally activate macrophages to become TBMs in the follicle

**Figure 7:**
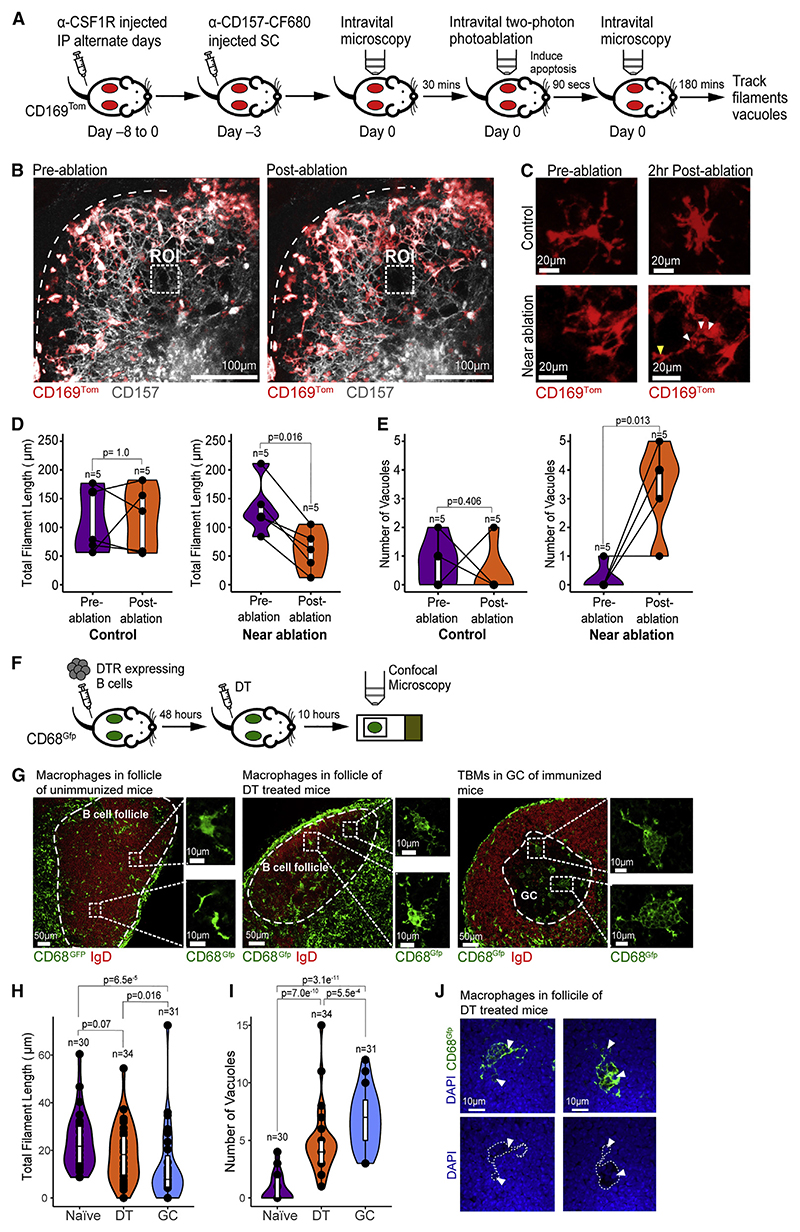
Nearby apoptotic cells activate TBM maturation (A) Schematic for localized induction of B cell apoptosis in an ROI in the primary follicle by intravital two-photon photoablation in CD169^Tom^ mice. (B) MIP of a lymph node showing the follicular stroma labeled with anti-CD157 (gray) and follicular macrophages (red) before (left panel) and after (right panel) intravital two-photon photoablation of the ROI. (C) Time-lapse images of follicular macrophages distal to (top panels) and neighboring (bottom panels) the ROI before (left panels) and 2 h after (right panels) intravital two-photon photoablation. Arrowheads indicate vacuoles. (D) Violin plot of total filament length pre- and post-ablation for control macrophages distal to (left panel) and macrophages near the ROI (right panel). n = number of macrophages tracked. (E) Violin plot of number of vacuoles pre- and post-ablation for control macrophages distal (left panel) and macrophages near the ROI (right panel). n = number of macrophages tracked. (F) Schematic for inducible ablation of adoptively transferred B cells by diphtheria toxin in CD68^Gfp^ mice. (G) MIP confocal images showing macrophages in the primary follicles of unimmunized mice (left panel) and diphtheria toxin (DT) treated mice (middle panel) and secondary follicles of day 7 CGG/AddaSO3 immunized mice (right panel), stained for IgD(red)-negative GCs. Representative of results from three mice per group and two experiments. (H) Violin plot of total filament length for GFP+ macrophages in the follicles of naive, diphtheria toxin- (DT) treated mice and in the GC of immunized mice. (I) Violin plot of number of vacuoles for GFP + macrophages in the follicles of naive, diphtheria toxin- (DT) treated mice and in the GC of immunized mice. (J) Confocal image showing macrophages in the primary follicle of DT-treated mice stained with DAPI (blue). Arrows point to DAPI+ DNA within vacuoles.

The finding of TBM-like cells in the absence of GCs suggests that factors other than antigens or adjuvants may activate their transition to classical TBMs. We tested the hypothesis that dead cells themselves may locally activate TBMs by inducing B cell apoptosis by intravital two-photon excitation (Figure 7A). We first depleted SSMs with CSF1R blockade to ensure that we were investigating follicular macrophages and also to prevent signals from TOM+ SSMs interfering with the image analysis. The follicular stroma was labeled with anti-CD157-CF680 monoclonal antibody, and regions of interest (ROI) in the follicle were irradiated in a targeted manner with NIR light to induce B cell apoptosis^45^ (Figure 7B; Video S6). Ablation of the CF680 signal in the ROI was used as a marker of photodamage (Figure 7B). Intravital imaging revealed changes in the behavior of the macro-phages bordering the ROI, with increased scanning by processes that reached into the ROI (Video S6). In contrast to the control macrophages, within 2 h, these macrophages accumulated more vacuoles and had shorter cell processes (Figures 7C–7E). Next, we used *Mb1^Cre/+^.Rosa26^iDTR^* (B cell-specific DTR) mice in which B cells can be inducibly ablated by the administration of diphtheria toxin.^46^ B cells from these mice were adoptively transferred into CD68^Gfp^ recipient mice and allowed 48 h to home to the lymph nodes and equilibrate before diphtheria toxin was injected (Figure 7F). Analysis of the lymph nodes 10 h later revealed the accumulation of multiple phagocytic vacuoles in TBM-like cells (Figures 7G–7I). Some of these vacuoles contained DAPI + DNA from phagocytosed nuclei, confirming that they contained apoptotic B cells (tingible bodies) that had been targeted by diphtheria toxin treatment (Figure 7J). Notably, there were no GCs in the follicles as demonstrated by IgD staining, in comparison to TBMs in IgD-negative GCs of immunized mice (Figure 7G). These data provide evidence that TBM precursors can be locally activated by the presence of dead cells to acquire the classical features of a GC TBM in secondary follicles.

## Discussion

The silent, non-inflammatory disposal of apoptotic cells, termed efferocytosis, is a fundamental biological process that is required for embryonic development, organogenesis, tissue homeostasis, lymphocyte development, and immune function.^19,47^ Failure to remove apoptotic cells results in secondary necrosis,^48,49^ loss of membrane integrity, and release of immunostimulatory danger-associated molecular patterns (DAMPs).^49^ In the T cell zone, this function is performed by specialized CX3CR1^+^MERTK^+^ T cell zone macrophages (TZMs).^50^ In the B cell follicle, TBMs are similarly tasked with the removal of apoptotic cells in GCs. Here, we have used multiple redundant and complementary mouse reporter systems and lineage-tracing tools to determine the origin and function of TBMs. We show that CD169-lineage, CSF1R-blockade-resistant macrophages that are prepositioned in the follicle can be locally activated by the presence of dead cells to become TBMs in the absence of GCs. Our data reveal novel aspects of TBM biology in the lymph node that most likely reflect the adaptation and functional specialization of tissue-resident macro-phages^51–53^ that may be applicable to other sites of intense programmed cell death, such as the thymus.

Intravital microscopy revealed several unexpected findings. We observed GC B cells undergoing apoptosis in close proximity to TBMs before their subsequent capture and engulfment. While efferocytosis is thought to involve the engulfment of whole dying cells rather than dead cell fragments, we did not observe this, although we cannot exclude the possibility. This is surprising, because electron microscopy studies of the mouse spleen describe TBMs as containing the ingested corpses of whole dead cells identified as plasma cells.^10^ The electron micrographs were obtained of TBMs in the dark zone, and it is possible that we would have observed efferocytosis of intact cells if we had been able to penetrate deeper into the follicle. Similar observations of GC B cell fragmentation have been made from imaging of GC dynamics.^3,7^

We showed that TBMs were fixed in position and uniformly dispersed throughout the GC. In contrast, apoptotic cell fragments were able to migrate at a reduced speed in a “zombie” mode. It is possible that non-fluorescent cells could “bump” the fragments and give the impression of autonomous cell movement, or that apoptotic fragments may be transported by other GC cells such as B and T cells. Under the first scenario, we would expect the log(MSD) vs log(time) plot to show that apoptotic cells display random Brownian motion with α = 1.0, and under the second scenario, we would expect them to display similar motility to the GC B cells or T cells. However, our analysis shows that they are clearly subdiffusive with a slope a α 1, compared to GC B and T cells that were superdiffusive with a α 1.^31,32^ Interestingly, motile apoptotic cells have also been described in the developing zebrafish brain.^54^ We routinely observed non-migratory TBMs extending cytoplasmic processes to chase and capture dead cell fragments. Similar “lazy” search behaviors have been described for other tissue-resident macrophages, which may be dispersed due to territoriality from niche competition and mutual repulsion.^55^ These include Langerhans cells that perform dendrite surveillance extension and retraction cycling habitude (dSEARCH),^56^ liver capsular macrophages,^57^ and microglia.^58^

We next used a number of cell-fate-mapping tools and methods to show that nearly all TBMs originated from lymph node-resident cells, and a small minority originated from circulating blood monocytes. CSF1R blockade showed that TBMs do not derive from SSMs or MSMs. This is consistent with data showing that TBMs are CSF1R-blockade-resistant.^39,44^ TOM+ macrophages in CD169^Tom^ and GFP+ macrophages in CX3CR1^Gfp^ and CD68^Gfp^ mice were present in the primary follicles of unimmunized mice. While these cells did not have the classical appearance of TBMs, several contained the occasional phagocytic vacuole, suggesting that there was some low-level homeostatic cell death occurring in the follicles. Accordingly, the follicles may be the graveyards where senescent naive B cells go to die. These data indicated that macrophages in the follicles, distinct from SSMs, may also give rise to TBMs. This was confirmed by inducing B cell apoptosis by intravital two-photon photoablation,^45^ which locally activated these follicular macrophages to acquire the morphological features of TBMs. In complementary experiments, we induced more widespread death of adoptively transferred B cells and showed that after 10 h, the follicular macrophages had accumulated more vacuoles and shorter processes. These TBM-like cells were generated under conditions in which there were no GCs, suggesting that the presence of apoptotic cells and their DAMPs is sufficient to activate follicular macrophages to become TBMs. EdU labeling suggested that TBMs can undergo proliferative expansion. However, we do not exclude the possibility that TBMs can also arise from other lymph node-resident monocyte/macrophage sources outside the follicle.

CITE-seq resolved putative TBM cells based on their expression of *Mertk, Cx3cr1*, and *Cd68*. Putative TBMs were enriched for genes involved in apoptotic cell clearance, such as the C1Q family of soluble defense collagens also expressed by tissue-resident macrophages.^59,60^ Inherited C1Q deficiency is associated with impaired apoptotic cell clearance and SLE-like illnesses.^61^ Putative TBMs resemble TZMs, which also express *Mertk, Cx3cr1*, and *Cd68*.^50^ This may relate to the functional specializations of both tissue-resident macrophage populations. However, TZMs do not express CD169 protein and are not known to derive from a CD169-lineage precursor. Putative TBMs express *Clec9a* and therefore are developmentally distinct from TZMs, which do not derive from a CLEC9A+ precursor.^50^ Nevertheless, we cannot exclude the possibility that the scRNA-seq data is unable to resolve these two closely related populations.

Plasticity is a hallmark of macrophages, and it is likely that epigenetic programs, transcription factors, non-coding RNAs, and other regulatory mechanisms are involved in maintaining their diversity, adaptability, and function.^62^ The transcriptional cell state of macrophages may be more fluid and less discrete than that of other cell types. Accordingly, it may be difficult to identify classic cell state markers due to the heterogeneity, particularly for TBMs at different states of maturation. This contributes to the poor correlation between mRNA and protein expression in macrophages, which has been demonstrated for the *Csf1r* gene, where mRNA expression by TBMs in *Csf1r-*GFP mice^38^ is not matched by protein expression in CSF1R-FusionRed mice.^39^ The cleavage/shedding and secretion of proteins of interest, such as MERTK^63^ and CD206,^64^ and the C1Q family of complement proteins, respectively, add to the technical challenges of detecting potential TBM marker proteins. A study of CD206 expression from embryogenesis to adulthood reported that TBMs in unimmunized mice did not express CD206.^65^ However, we were able to detect CD206 by both FACS and confocal microscopy in a subset of TBMs. Transition of putative TBMs from the resting to immunized cell states is associated with the upregulation of a number of genes and biological processes that may facilitate the sensing, capture, and phagocytosis of cell fragments by cytoplasmic processes. While these data are consistent with the cells being TBMs, they are not conclusive, and it will be interesting to see future studies exploring the joint genomic, epigenomic, and proteomic regulation of TBM behavior.

There are still many unresolved aspects of TBM biology. For example, TBMs express high levels of class II MHC and have been shown to present antigens and interact with T cells *in vitro* via the production of prostaglandins,^66^ and it will be interesting to define these cell-cell interactions *in vivo* in real time. What, if any, other functions are served by TBMs? GC B cells outnumber TBMs by at least two orders of magnitude.^66^ If as many as half the number of GC B cells die every 6 h,^3^ then each TBM would need to remove 175–225 dead cells in the same time period. It is hard to conceive how TBMs could completely clear such an extraordinary load of debris in such a short time. Furthermore, as the GC reactions progress, TBMs undergo phenotypic maturation and accumulate more vacuoles, which may impair their capacity to take up and process more cellular debris. Thus, TBMs may be much more complex than previously appreciated, and it is hoped that the data presented here will provide the groundwork for future research into these enigmatic cells and their roles in health and disease.

## Limitations of the study

There are several challenges to studying rare immune cell populations, particularly those that are difficult to isolate from solid tissue.^67^ For tissue-resident macrophages, this is compounded by the risk of cell fragmentation,^68,69^ inadvertent cellular activation, and alteration of cell phenotypes and transcriptional states.^59^ Another limitation of scRNA-seq of macrophages is their plasticity and the likelihood that they exist in a fluid continuum of cell transcriptional states.^62^ This contrasts, for example, with naive B cell differentiation, which irreversibly occurs through linear steps via GC B cell, memory B cell, and plasma cell stages that represent discrete cell states. Macrophage plasticity also contributes to their heterogeneity and the poor correlation between mRNA and protein expression, for example, as has been documented for *Csf1r*.^70^ This has also been shown in a recent study by using joint single-cell transcript and protein profiling of heterogeneous macrophage cell states^71^ and implicates post-transcriptional regulation mechanisms.^72^ Future single-cell spatial and multiomic approaches may help to resolve this issue. In this study, we captured the cell states of CD11b^+^TOM^+^ cells from different lymph nodes, separated by 9 days, and it is likely that we did not capture the intermediate cell states along the continuum that bridge the origin (unimmunized) and terminal cell states (9 days post-immunization). Furthermore, TZMs^50^ and SSMs^73^ are seeded *in utero* and slowly replaced by a post-natal wave of bone marrow-derived monocytes. Future studies will be needed to determine the exact ontogeny of TBMs. Another limitation of the study is the penetration depth limit to intravital microscopy.^74^ As a result, it was not possible to penetrate through the entire B cell follicle to image the T cell zone, and we cannot completely rule out the possibility that some TBMs may migrate in from there. Future studies may be possible using adaptive optics or similar technologies to visualize the maturation and dispersion of TBMs in the nascent GC at high temporal resolution over longer time scales.

## Star Methods

### Key Resources Table

**Table:** 

REAGENT or RESOURCE	SOURCE	IDENTIFIER
Antibodies		
Anti-mouse CD169 purified (Siglec1)	This paper	N/A
Anti-mouse CD169 eFluor660 (Siglec1)	Invitrogen	Cat #: 50-5755-82; RRID:AB_2574241
Anti-mouse CD68-Biotin (FA-11)	Bio-Rad	Cat #: MCA1957BT; RRID:AB_323443
Anti-GFP (polyclonal)	ThermoFisher	Cat #:A-11122; RRID:AB_221569
Anti-mouse IgD BV421 (11-26c.2a)	BioLegend	Cat #: 405,725; RRID:AB_2562743
Anti-mouse CD2⅓ 5 APC (7E9)	BioLegend	Cat #: 123,412; RRID:AB_2085160
Anti-mouse/human GL7 PB (GL7)	BioLegend	Cat #: 144,614; RRID:AB_2563292
Anti-mouse Tim-4 (RMT4-54)	BioLegend	Cat #: 130,002; RRID:AB_1227802
Donkey anti-rabbit IgG AF488	ThermoFisher	Cat #: A-21206; RRID:AB_2535792
Goat anti-rat IgG AF555	ThermoFisher	Cat #: A-21434; RRID:AB_2535855
Anti-mouse CD3 APC Cy7 (145-2C11)	BioLegend	Cat #: 100,330; RRID:AB_1877170
Anti-mouse CD4 APC Cy7 (GK1.5)	BioLegend	Cat #: 100,414; RRID:AB_312699
Anti-mouse CD19 APC Cy7 (6D5)	BioLegend	Cat #: 115,530; RRID:AB_830707
Anti-mouse CD8 APC Cy7 (53-6.7)	BioLegend	Cat #: 100,714; RRID:AB_312753
Anti-mouse CD45.2 AF647 (104)	BioLegend	Cat #: 109,818; RRID:AB_492870
Anti-mouse SiglecF BV605 (E50-2440)	BD Biosciences	Cat #: 740,388; RRID:AB_2740118
Anti-mouse/human CD11b BV785 (M1/70)	BioLegend	Cat #: 101,243; RRID:AB_2561373
Anti-mouse CD115 AF488 (CSF-1R)	BioLegend	Cat #: 135,512; RRID:AB_11218983
Anti-mouse Ly6C PE Cy7 (HK1.4)	BioLegend	Cat #: 128,018; RRID:AB_1732082
Anti-mouse Ly6G APC Cy7 (1A8)	BioLegend	Cat #: 127,624; RRID:AB_10640819
Anti-mouse CD3-FITC (500A2)	Invitrogen (eBioscience)	Cat #: 11-0033-82; RRID:AB_837091
Anti-mouse CD11b-Percpcy5.5 (M1/70)	Invitrogen (eBioscience)	Cat #: 45-0112-82; RRID:AB_953558
Anti-mouse F4/80-BV660 (BM8)	BioLegend	Cat #: 123,149; RRID:AB_2564589
Anti-mouse CD4-BP (RM4-5)	BD Pharmingen	Cat #: 558,107 RRID:AB_397030
Anti-mouse V α2-FITC (B20.1)	eBioscience	Cat #: 11-5812-82 RRID:AB_465259
Anti-mouse B220-PB (RA3-6B2)	BD Pharmingen	Cat #: 558,108 RRID:AB_397031
HyHEL9-A647	This paper	N/A
Anti-mouse B220-Biotin (RA3-bB2)	BD Biosciences	Cat #: 553,086; RRID:AB_394616
Anti-mouse CD11b-Biotin b220 (M1/70)	BD Biosciences	Cat #: 557,395; RRID:AB_2296385
Anti-mouse CD11c-Biotin (HL3)	BD Biosciences	Cat #: 553,800; RRID:AB_395059
Anti-mouse CD8 a-Biotin (53-6.7)	BioLegend	Cat #: 100,703; RRID:AB_312742
Anti-mouse Ly6G-Biotin (1A80	BioLegend	Cat #: 127,603; RRID:AB_1186105
Anti-mouse CD43-Biotin (S7)	BD Biosciences	Cat #: 553,269; RRID:AB_2255226
Anti-mouse CD4-Biotin (GK1.5)	BD Biosciences	Cat #: 553,728; RRID:AB_395012
Anti-Ki67 AF647	BioLegend	Cat #: 151,206; RRID:AB_2566801
Anti-mCherry	Thermo Fisher Scientific	Cat #: M11217
Anti-rat AF555	Thermo Fisher Scientific	Cat #: A21434
Anti-mouse MERTK-BV786 (DS5MMER)	Invitrogen (Thermo Fisher)	Cat #:78-5751-82 RRID: AB_2762814
Anti-mouse CD11b-Buv395 (M1/70)	BD Bioscience (Horizon)	Cat #:565,976 RRID: AB_2721166
Anti-mouse CX3CR1-BV78 (SA011F11)	BioLegend	Cat #: 149,029 RRID: AB_2565938
Anti-mouse CD206-biotin (C068C2)	BioLegend	Cat #: 141,713 RRID: AB_10918434
SA-BUV395	BD Bioscience (Horizon)	Cat #: 564,176 RRID: AB_2869553
Normal mouse serum	Jackson ImmunorResearch (Abacus Dx)	Cat #:015-000-120
Avidin/Biotin Blocking Kit	Thermo Fisher Scientific	Cat #: 004,303
Anti-mouse Fc block (2.4G2) anti-mCD16/32	BioXCell	Cat #: 4364B/1013
Anti-CD157 purified (BP3)	This paper	N/A
Anti-mouse CSF1R (AFS98)	BioXCell	Cat #: BE0213; RRID:AB_2687699
Anti-mouse IgG2a (C1.18.4)	BioXCell	Cat #: BE0085; RRID:AB_1107771
TotalSeq-A0106 anti-mouse CD11c	BioLegend	Cat #: 117,355; RRID:AB_2750352
TotalSeq-A0015 anti-mouse Ly-6G	BioLegend	Cat #: 127,655; RRID:AB_2749962
TotalSeq-A0013 anti-mouse Ly-6C	BioLegend	Cat #: 128,047; RRID:AB_2749961
TotalSeq-A0440 anti-mouse CD169 (Siglec-1)	BioLegend	Cat #: 142,425; RRID:AB_2783106
TotalSeq-A0560 anti-mouse CD68	BioLegend	Cat #: 137,031; RRID:AB_2783099
TotalSeq-A0301 anti-mouse Hashtag 1	BioLegend	Cat #: 155,801; RRID:AB_2750032
TotalSeq-A0302 anti-mouse Hashtag 2	BioLegend	Cat #: 155,803; RRID:AB_2750033
TotalSeq-A0303 anti-mouse Hashtag 3	BioLegend	Cat #: 155,805; RRID:AB_2750034
TotalSeq-A0304 anti-mouse Hashtag 4	BioLegend	Cat #: 155,807; RRID:AB_2750035
Chemicals, peptides, and recombinant proteins		
DAPI	Sigma Aldrich	Cat #: D9542
OVA_323-339_ peptide	Mimotopes or GenScript	N/A
Lysozyme from chicken egg white (HEL)	Sigma Aldrich	Cat #: L6876-1G
Sigma Adjuvant System	Sigma Aldrich	Cat #: S6322-1VL
Formalin solution, neutral buffered, 10%	Sigma Aldrich	Cat #: HT5011-15mL
Xylazine	Troy Laboratories	Cat #:XYLAZ 2
Ketamine	Mavlab Animal Health	Cat #: KETA M I
Buprenorphine (Temgesic)	Sigma	Cat #: TEMG I
T-putty	Thermagon Inc	Cat #: A10548-5
Silicone Grease	Beckman Coulter	Cat #: 335,148
EDTA	Sigma	Cat #: E5134
RPMI 1640 Medium, GlutaMAX™ Supplement	Gibco/ThermoFisher Scientific	Cat #: 61,870,036
PBS, pH 7.4	Gibco/ThermoFisher Scientific	Cat #: 10,010,049
Fetal Bovine Serum, heat inactivated	Bovogen	Cat #: SFBS
O.C.T Compound	Sakura	Cat #: 4583
Fluoromount-G	Southern Biotech	Cat #: 0100-01
Fetal Bovine Serum, qualified, heat inactivated	GIBCO	Cat #: 10,500,056
Fetal Bovine Serum	GIBCO	Cat #: 10,500,064
Penicillin and Streptomycin	Thermo Fisher Scientific	Cat #: 15,140,122
Normal rat serum	Thermo Fisher Scientific	Cat #: 31,888; RRID: AB_2532178
Normal donkey serum	Sigma Aldrich	Cat #: D9663-10ML
Paraformaldehyde	Thermo Fisher Scientific	Cat #: 043,368.9M
Chicken Gamma Globulin	Rockland	Cat #: D602-0100
AS03-like adjuvant	InVivoGen	Cat #: vac-as03-10
Tamoxifen free base	Merck Life Science	Cat #:T5648-1G
Corn Oil	Sigma Aldrich	Cat #: C8267
Diphtheria toxin	Calbiochem, Merck	Cat #: 322,326
Baytril	Bayer	N/A
Prolong Diamond Antifade	Thermo Fisher Scientific	Cat #: P36965
Triton X-100	Merck	Cat #: X100
Sucrose	Sigma Aldrich	Cat #: S0389-500G
100% Acetone	Thermo Fisher Scientific	Cat #: 177,170,050
Liberase TM Research grade	Roche	Cat #: 5,401,119,001
DNasel	Sigma	Cat #: 10,104,159,001
CF680 conjugation kit	(Biotium)	Cat #: 29,070 - 29,082
Click-iT EdU Cell Proliferation Kit for Imaging, Alexa Fluor™ 647 dye	Thermo Fisher Scientific	Cat #:C10340
5-Ethynyl-2′-deoxyuridine (EdU)	Biosynth Carbosynth	Cat #: NE08701
Critical commercial assays		
Chromium Single Cell 3′ GEM, Library & Gel Bead Kit v3.0	10X Genomics	Cat #: 1,000,092
Chromium Chip B Single Cell Kit	10X Genomics	Cat #: 1,000,074
Chromium i7 Multiplex Kit	10X Genomics	Cat #: 120,262
NovaSeq 6000 S1 Reagent Kit v1.0 (300 cycles)	Illumina	Cat #: 20,012,863
Deposited data		
CITE-Seq data	GEO Datasets	GSE214060
Experimental models: Organisms/strains		
C57BL/6-Tg(CD68-EGFP)1Drg/J mice	Jackson Labs	JAX: 026,827
B6.129P2(C)-Cx3cr1tm2.1(cre/ERT2)Jung/J mice	Jackson Labs	JAX: 020,940
B6; 129S-Gt(ROSA)26Sortm1.1Ksvo/J	Jackson Labs	JAX: 023,139
B6.129P2(Cg)-Cx3cr1tm1Litt/J mice	Jackson Labs	JAX: 005,582
Cd79atm1(cre)Reth mice	Jackson Labs	JAX: 020,505
B6.Cg-Gt(ROSA)26Sortm9 (CAG-tdTomato)Hze mice	Jackson Labs	JAX: 007,914
Gt(ROSA)26SorFlox.stp.DTR mice	Jackson Labs	JAX: 007,900
B6SJL CD45.1 mice	Oxford University core breeding facility	N/A
C57/BL6J CD45.2 mice	Oxford University core breeding facility	N/A
C57BL/6N-Ccr2tm1(cre/ERT2, mKate2)Arte/BechJ	Burkhard Becher, University of Zurich, Switzerland	^91^
C57BL/6-Siglec1<tm1(cre)Mtka>	Masato Tanaka, Tokyo University of Pharmacy and Life Sciences, Japan; BRC	RBRC06239
B6.Cg-Gt(ROSA)26Sor < tm1.1(CAG-kikGR)Kgwa>	BRC	RBRC04847
Tg(CAG-Kaede)#Kgwa	Michio Tomura, Osaka Ohtani University	N/A
SWHEL mice	Robert Brink, Australian BioResources	N/A
Tg(ACTB-ECFP)1Nagy/J	Jackson Labs	JAX: 003,773
B6.Cg-Tg(TcraTcrb425Cbn/J	Jackson Labs	JAX: 004,194
Software and algorithms		
CellRanger 4.0.0	10X Genomics	https://support.10xgenomics.com
CITE-seq-count	Hobeika et al.^81^	https://github.com/Hoohm/CITE-seq-Count
R 4.1.0	The Comprehensive R Archive Network	https://cran.r-project.org/
RStudio 1.4.1717	RStudio	https://www.rstudio.com/
Seurat_4.1.0	Phan et al.^82^	https://github.com/satijalab/seurat
NMF_0.24.0	Tomura et al.^83^	http://renozao.github.io/NMF/
swne_0.6.2	Sasmono et al.^38^	https://github.com/yanwu2014/swne
edgeR	Hadjantonakis et al.^84^	https://bioconductor.org/packages/edgeR/
GSEA 4.2.3	Barnden et al.^85^	https://www.gsea-msigdb.org/gsea
M5.all.v0.3.symbols.gmt	msigDB	https://www.gsea-msigdb.org/gsea/msigdb
Cytoscape 3.9.1	Stoeckius et al.^86^	www.cytoscape.org
EnrichmentMap 3.3.4	Luke et al.^87^	http://www.baderlab.org/Software/EnrichmentMap
AutoAnnotate 1.3.5	Read et al.^88^	http://www.baderlab.org/Software/AutoAnnotate
FIJI	Read et al.^89^	https://imagej.net/Fiji
QuPath	90	https://qupath.github.io/
Adobe Photoshop	Adobe	https://www.adobe.com/au/products/photoshop.html
Adobe After Effects	Adobe	https://www.adobe.com/au/products/aftereffects/campaign/pricing.html
Adobe Illustrator CC	Adobe	https://www.adobe.com/uk/products/illustrator.html
Imaris, 9.8.9	Oxford Instruments	https://imaris.oxinst.com/versions/9-8
MATLAB	MathWorks	https://www.mathworks.com/products/matlab.html
Flowjo, version 10	Tree Star	https://www.flowjo.com/
Prism, version 9	GraphPad	https://www.graphpad.com/scientific-software/prism/; RRID:SCR_002798
FACSDiva	BD Biosciences	http://www.bdbiosciences.com/us/instruments/clinical/software/flow-cytometry-acquisition/bd-facsdiva-software/m/333333/overview
Java JRE 17.0.1	Oracle	https://www.oracle.com/java/technologies/javase/jdk17-archive-downloads.html
Python 3.9	Python	https://www.python.org/downloads/release/python-390/
Mathematical modeling	GitHub	https://github.com/theimagelab/tbm https://doi.org/10.5281/zenodo.7587414
BioRender	BioRender	https://biorender.com/
Other		
Nil		

## Resource Availability

### Lead contact

Further information and requests for resources and reagents should be directed to and will be fulfilled by the Lead Contact, Dr Tri Giang Phan (t.phan@garvan.org.au).

### Materials availability

This study did not generate unique reagents.

## Experimental Model and Subject Details

Animal experiments were performed in accordance with approved protocols from Garvan Institute/St Vincent’s Hospital Animal Ethics committee (ARA 18/33 and 2½5). All experiments were performed under the authorization by a project license granted by the UK Home Office and were also approved by the Institutional Animal Ethics Committee Review Board at the University of Oxford.

### Animals

CD169^Tom^ mice were generated by crossing CD169-Cre mice (RBRC06239; C57BL/6-Siglec1<tm1(cre)Mtka>)^29^ with mice carrying the LSL-tdTomato mice (007,914; B6.Cg-Gt(ROSA)26Sortm14(CAG-tdTomato)Hze/J).^75^ CD169^Kik^ mice were generated by crossing CD169-Cre mice with floxed Kikume mice (RBRC04847; B6.Cg-Gt(ROSA)26Sor < tm1.1(CAG-kikGR)Kgwa>).^76^ CD68^Gfp^ transgenic mice (026,827; C57BL/6-Tg(CD68-EGFP)1Drg/J)^77^ and CX3CR1^Gfp^ knock-in/knock-out mice (005,582; B6.129P2(Cg)-Cx3cr1tm1Litt/J)^78^ have been previously described. *Cx3cr1*^CreER/+^*.Rosa*^lsl-dTom/+^ mice were generated by crossing Cx3cr1-CreER mice (020,940; B6.129P2(C)-Cx3cr1tm2.1(cre/ERT2)Jung/J)^79^ to LSL-tdTomato mice. *Ccr2*^CreER/+^.*Rosa*^lsl-dTom/+^ mice were generated by crossing Ccr2-CreER mice (Ccr2tm1(cre/ERT2,mKate2)Arte)^80^ to LSL-tdTomato mice. Mb1-DTR transgenic mice were generated by crossing CD79a-CreERT2 mice (033,026; B6.C-Cd79atm3(cre/ERT2)Reth/EhobJ)^81^ to ROSA26-DTR mice (007,900; C57BL/6-Gt(ROSA)26Sortm1(HBEGF)Awai/J).^46^ SW_HEL_ mice expressing a knock-in BCR against hen egg lysozyme (HEL)^82^ were maintained on a C57BL/6J or C57BL/6-SJL.Ptprc^a/a^ congenic background. Kaede transgenic mice (Tg(CAG-Kaede)#Kgwa)^83^ were crossed to SW_HEL_ mice to obtain green fluorescent SW_HEL_ B cells on a C57BL/6J background. CFP transgenic mice expressing cyan fluorescent protein under the β–actin promoter (003,773; Tg(ACTB-ECFP)1Nagy/J)^84^ were crossed to OT-II TCR transgenic mice (004,194; B6.Cg-Tg(TcraTcrb425Cbn/J),^85^ and maintained on a C57BL/6 background. C57BL/6 and C57BL/6-SJL.Ptprc^a/a^ congenic mice were purchased from Australian BioResources (Moss Vale, Australia).

All mice were bred and maintained on a C57BL/6J background, were maintained in individually ventilated cages and were at over 6 weeks of age at the time of experimentation. Both males and females were used. Mice were housed at the Australian BioResources, the Garvan Institute of Medical Research Biological Testing Facility or the University of Oxford Biomedical Sciences facility under pathogen free conditions and fed standard mouse chow. Animal experiments were performed under the approved guidelines of the Garvan Institute of Medical Research and St Vincent’s Hospital Animal Ethics Committee and by a project licence granted by the UK Home Office and by the Institutional Animal Ethics Committee Review Board at the University of Oxford. Mice were genotyped by the Garvan Molecular Genetics facility at the Garvan Institute of Medical Research or at the University of Oxford.

## Method Details

### Bone marrow chimera generation

To generate bone marrow chimeric mice, mice were lethally γ-irradiated at 4.5 Gy for 300 s, followed by a 3-h rest period, and a sub-sequent 4.5 Gy dose for 300 s. For shielded irradiation, isoflurane anesthetized mice were irradiated from an X-ray source with two doses of 5.5 Gy for 232 s, separated by 3-h rest period. During irradiation, the right inguinal lymph node was shielded with a lead block that was measured to reduce radiation exposure by 92%. Following irradiation mice were injected i.v. with donor bone marrow and provided with antibiotics (0.16 mg/mL Enrofloxacin (Baytril) in drinking water for 4 weeks. Mice were allowed to reconstitute for a minimum of eight weeks.

### Adoptive cell transfer and immunizations

Donor mice were euthanised by CO_2_ inhalation and cervical dislocation and the spleens harvested. Single cell suspensions of splee-nocytes were prepared by mashing spleens through a 70μm nylon mesh filter over a 50mL Falcon tube with a 1mL syringe plunger end. Centrifugation steps in 15mL or 50mL tubes were spun at 1500rpm (270 rcf), 5 min, 4°C. Red blood cells (RBCs) were lysed using 4mL 31 RBC lysis buffer under-layered with 1mL Fetal Bovine Serum (FBS). Cells were washed in 10mL FACs wash and resuspended at 10^8^ cells/mL. OT-II cells were enriched by negative depletion with biotinylated antibodies for anti-B220, anti-CD11b, anti-CD11c, anti-Ly6G anti-CD8a. Naive B cells and SW_HEL_ B cells were enriched by negative depletion with biotinylated antibodies for anti-CD4, anti-CD11b, anti-CD11c, anti-CD43 and detected with MACs anti-biotin magnetic beads (Miltenyi). Washed and filtered cells were passed through LS columns on a MACs Separator to isolate the negative fraction donor population of interest. Purity of CD4^+^ Vα2^+^ OT-II population or B220^+^ HEL-binding SW_HEL_ population was determined by FACs analysis. Cells were filtered through 35μm filter round-bottom FACs tubes (Corning) prior to data acquisition on either LSR II or Fortessa (BD Biosceiences). 2.5 × 10^5^ CD4+Vα2^+^ Cyan OT-II T cells and Kaede B220^+^ HEL-binding B cells were injected intravenously into age and sex matched recipients. At times, for clearer imaging, 2.5 × 10^5^ CD4+Vα2^+^ Cyan OT-II T cells, 0.5 × 10^5^ Kaede B220^+^ HEL-binding B cells and 2.0 × 10^5^ WT B220^+^ HEL-binding B cells were injected intravenously into age and sex matched recipients. For naive B cell transfer studies, 10 million naive B cells were injected intravenously into age and sex matched recipients. For B cell ablation studies, RBC lysed unfractionated splenocytes from single donor Mb1-DTR mice spleen were transferred intravenously per recipient.

### Adjuvant based antigen immunization

Recipient mice of the OT-II cells and SW_HEL_ B cells were immunized the day after cell transfer. This was done subcutaneously with 20μg HEL-OVA in Sigma Adjuvant System (SAS) (Sigma) in the upper and lower flanks and at the tail base of mice.

### HEL-OVA antigen preparation

To prepare HEL-OVA, HEL was chemically conjugated to OVA_323-339_ peptide (CGGISQAVHAAHAEINEAGR) (Mimotopes or Genscript) using the cross-linking agent Succinimidyl-6-([β-maleimidopropionamido] hexanoate) (SMPH) (Thermo Fisher Scientific).

### CGG immunization

To generate a localised immune response, mice were immunized subcutaneously with 10μg of chicken gamma globulin (CGG) mixed with equal volume of AS03-like adjuvant (AddaS03, Invivogen) into the upper and lower flanks (100μL per animal side).

### CSF1R blocking

200μg rat anti-mouse CD115 antibody (BioXCell) or rat anti-mouse IgG2a (BioXCell) was administrated via intra-peritoneal injection every-other day for a total of 4 doses in resting mice or a total of 9 doses in immunized mice. The anti-CD115 antibody dose was determined as described previously.^44^

### Diphtheria toxin treatments

Mice received single doses of 0.75μg diphtheria toxin (Calbiochem, Merck) in saline by i.p. injection.

### Tamoxifen treatments

Mice were injected intraperitoneally with 2mg of tamoxifen dissolved in 10% ethanol, 90% corn oil.

### Preparation of single cell suspensions

Mice were euthanised by CO_2_ inhalation and cervical dislocation and organs of interest were harvested and processed into single cell suspensions at a staining concentration of 1.5×10^7^ cells/mL.

### Lymph node digestion

4-6 lymph nodes (inguinal, auxiliary and brachial) were dissected per mouse. The lymph nodes were teased apart with dissecting forceps and digested in 200μg/ml Liberase (Roche) and 20μg/ml DNase (Sigma) for 20 min at 37°C in PBA (PBS with 0.1% BSA, 0.02% sodium azide). Digestion was inactivated by adding 500μL FBS and 25μL (0.5M) EDTA. The digested lymph nodes were then mashed through a 100μm nylon mesh cell strainer and washed three times with PBA. Centrifugation steps were spun at 1500rpm (270 rcf), 5 min, 4°C.

### FACS analysis

Single-cell suspensions were prepared as described above. Cells were blocked with Fc block (anti-CD16/32) prior to staining. To detect HEL-binding B cells for adoptive transfer, cells were stained with HEL at 200 ng/ml, followed by HyHEL9 Alexa Fluor 647. Antibodies used are shown in key resources table and were made up in FACS buffer. In-house conjugation was performed using a fluorescent protein labeling kit to CF680 (Biotium). Cells were filtered using 35μm filter round-bottom FACS tubes (BD Biosciences) immediately prior to data acquisition on either an LSR II SORP or Fortessa (BD Biosciences) and data analyzed using FlowJo software (Tree Star, Inc.).

### Cell sorting and 10X capture for scRNA-seq

9 days post-immunization, inguinal lymph nodes were dissected from each CD169^Tom^ mouse (2 unimmunized and 2 immunized). The lymph nodes were dissected and digested as described above and single cell suspensions were prepared. Cells were stained with CD11b-PerCP-Cy5.5, a panel of CITE-seq antibodies (as listed in the Reagents and Resources table), and one hashtag antibody per mouse, and then sorted on a FACSAria III (BD Biosciences) for CD11b^+^TOM^+^ cells. A total of 90,000 hash-tagged cells were sorted at >95% purity. These were combined into one multiplexed sample that was split across two Chromium 10X capture reactions. 18,662 single cells were encapsulated in Gel-Beads-in emulsion (GEMs) using the 10X Controller with a Chromium Single Cell 3′ GEM Library and Gel Bead kit (Garvan Weizmann Center for Cellular Genomics). The CITE-seq and hashing protocol described by Stoeckius et al. was used to generate an antibody-derived tag (ADT) sequencing library, a hashtag oligo (HTO) library and a 3′ gene expression library for each capture reaction.^86^ The libraries were sequenced on an Illumina NovaSeq 6000 (Ramaciotti Center for Genomics, UNSW Australia).

### Immunofluorescence microscopy

#### Tissue preparation

Lymph nodes were harvested and fixed in formalin solution 10% neutral buffered (Sigma-Aldrich), dehydrated in 30% sucrose gradient (Sigma) and frozen in optimal cutting temperature compound (OCT) (Sakura) in Tissue-Tek cryomolds (Sakura). 30μm or 10μm sections were cut on a cryostat and captured on Superfrost Plus glass slides (Thermo Scientific). 10μm section slides were further submerged in 100% ice-cold acetone (Thermo-Fischer scientific) and dried overnight before staining. Cut sections were blocked using the appropriate species blocking serum in 3% BSA in PBS. All antibodies used for staining sections are described in key resources table and were made up in 0.01% BSA in PBS. In house conjugation was performed using a fluorescent protein labeling kit to CF680 (Biotium). Staining mixes that contained fluorescent antibody mixes were spun down at full speed for 10 min before use. For 30 μm sections, 0.3% Triton X-100 (Merck), 0.2% BSA (Merck) and appropriate species blocking serum were included in staining solution. All staining steps were performed at room temperature for an hour, or 6 h to overnight at 4°C for 30μm sections with washing 33 between steps in PBS solution. Fluoromount G (Southern Biotech) or Diamond Prolong Antifade (Thermo Fisher Scientific) were used to mount coverslips (Menzel-Gläser) prior to visualisation of slides.

#### EdU labeling

Mice hemizygous for CX3CR1-CreERT2, Rosa26-LSL-mCherry and CD68-GFP were treated with tamoxifen by oral gavage (12mg) once followed by 3 additional i.p. tamoxifen injections (2mg) on days 2, 4 and 6. The animals were subsequently immunized s.c. with CGG/AddSO3 1 week later. Mice received EdU (0.45 mg/ml, supplemented with 2% sucrose) in their drinking water continuously from the day after immunization until tissue harvest on day 11. Fixed frozen tissue sections (40μm) were stained. Click-iT EdU staining was performed as per manufacturer’s instructions, except incubation was 1h 2% rat serum was included in all steps following anti-rat AF555.

#### Image acquisition

Images were collected on a Leica DMI 6000, SP8 Basic Confocal microscope, a Zeiss 780 confocal microscope, or a Zeiss Axioscan slide scanner. Images were acquired with a Plan-Apochromat 20×, 40×, 60× or ×100 oil immersion objective. Excitation lasers used were 405nm, 488nm, 554nm and 632nm and detectors were set according to the corresponding fluorophores used. Typically, images were acquired at 1024 × 1024 pixels, 100 Hz scan speed and with the pinhole set to 1 Airy unit. Leica LSX or ZEN2010 software was used to capture z stack or single images per fluorochrome. Images were compiled and brightness and contrast adjusted in Adobe Photoshop or ImageJ. Maximum intensity projections of z-stacks were created using ImageJ software. Tile images were collected on a Leica DM600 confocal microscope. LAS v4.5 software was used to capture single images per fluorochrome used for staining. Images were compiled and brightness and contrast adjusted in Adobe Photoshop or ImageJ.

#### Colocalization

A colocalization channel of CD169^Tom^ and CD206 was made in Imaris using the colocalization function. TBM and MSM were segmented using the surface tool, masked onto separate channels.

### Two-photon microscopy

#### Fiducial labeling for two-photon microscopy

The anti-CD157 monoclonal antibody clone BP-3 was conjugated with CF680 protein labeling kit (Biotium) and used to label follicular stroma (injected overnight) or follicular dendritic cell network (injected >3 days prior) as has been previously described.^30^

#### Explant lymph node imaging preparation

The mouse was euthanised by CO_2_ inhalation and cervical dislocation and the inguinal LN taken. The LN was separated from adipose and connective tissue under a stereomicroscope with low-level illumination. The LN was mounted, cortical side up, on a cut plastic coverslip with VetBond tissue glue. Excess glue was removed under the stereomicroscope, as VetBond glue is fluorescent and can interfere with imaging. Silicone grease was used to adhere the coverslip to a 6cm Petri dish.

#### Intravital lymph node imaging preparation

Anesthesia was induced in the mouse with 100 mg/kg ketamine/20 mg/kg xylazine and maintained with 0.5–1.5% isoflurane supplemented with 100% oxygen at a flow rate of 500 mL/min via a nose cone.^31^ The anesthetised mouse was kept warm on a customized heated SmartStage (Biotherm) set to 37°C and Lacri-Lube was applied to its eyes to prevent dryness. Hair on lower flank and inguinal area was shaved and micropore tape used to remove further hairs. Skin was sterilised with 70% ethanol and sterile scissors used to cut a skin flap on either the right-hand or left-hand flank that exposes the inguinal lymph node. T-putty (LairdTech) was applied to base of PDMS polymer (Dow Corning) and stuck to base of heating pad. VetBond tissue glue was used on the outside of PDMS to stick the skin flap down on to the PDMS base. Skin over the inguinal lymph node was cut with microdissection scissors and PBS applied to prevent the lymph node from drying out. Adipose tissue covering and surrounding the LN was removed under a stereomicroscope with low-level illumination. An O-ring was stuck down with silicone grease around the inguinal lymph node and PBS applied to create a meniscus. At times, Immersol (Carl Zeiss) was also applied to provide additional meniscus stability.

#### Photoconversion surgery

Anesthesia was induced with 100 mg/kg ketamine/20 mg/kg xylazine. The anesthetised mouse was kept warm on a customized heated SmartStage (Biotherm) set to 37°C and Lacri-Lube ointment was applied to its eyes to prevent dryness. Hair on lower flank and inguinal area was shaved and micropore tape used to remove further hairs. Skin was sterilised with 70% ethanol and sterile scissors used to cut a small incision to reveal the inguinal lymph node. Some of the surrounding adipose tissue was removed and the exposed lymph node was exposed to white light for 30 min. PBS was applied at regular intervals to the lymph node to prevent it from dying out. The outer skin was sutured and sham surgery was performed on the other inguinal lymph node. After recovery from anesthesia, 0.75 mg/kg of buprenorphrine was injected subcutaneously for post-operative analgesia. Mice were given gel food, monitored closely and sacrificed up to 7 days post-surgery.

#### Two-photon image acquisition

Imaging was performed on a Zeiss 7MP two-photon microscope (Carl Zeiss) powered by a Chameleon Vision II NIR laser (Coherent Scientific) tuneable between 690 and 1060nm. Images were acquired with a W Plan-Apochromat 20×/1.0 DIC (UV)Vis-IR water immersion objective. Excitation wavelength used was 920nm. Fluorescent images were acquired with an LBF 760 and BSMP 760 to enable detection of far-red signals. Non-descanned detectors (NDD) were SP 485 (blue; SHG and CFP), 50 BP0-550 (green; Kaede, CFP), 56 BP5-610 (red; Kikume red, CF555) and 64 BP0-710 (far-red; CF680). Typically, 75-120μm z-stacks, sampling every 3μm, were acquired. Image stacks were acquired at 30 s time intervals for 30-60-min movies for 60-120 cycles. Imaging was also performed on an FVMPE-RS two-photon microscope (Olympus) powered by InSight X3 (Spectra-Physics) with a tuning range of 680nm–1300nm and MaiTai DeepSee eHP (Spectra-Physics) with a tuning range 690nm–1040nm NIR lasers. Images were acquired at 930nm and 1140nm. Filter Sets for NDD include: violet (410nm–455nm) and green (495nm–540nm) fluorescence; cyan (460nm–500nm) and yellow (520nm–560nm) fluorescence; green (495nm–540nm) and red (575nm–645nm) fluorescence, 475 (465nm–485nm, for SHG) and red (605nm–685nm). We also used the FV30-FRCY5 with red barrier filter (575nm–645nm), 650nm dichroic mirror and Cy5 barrier filter (660nm–750nm) for red and far-red detection. Typically, 75-120μm z-stacks, at 3μm z-step intervals, were acquired. Image stacks were acquired at 30 s time intervals for 30-60-min movies for 60-120 cycles.

#### Intravital two-photon photoablation

Surgery was performed as above for **Intravital Lymph Node Imaging Preparation** and two-photon microscopy was performed on the FVMPE-RS two-photon microscope. The Region of Interest (ROI) was selected and targeted for intravital two-photon photoablation. The MaiTai laser was set to 800nm and 2.66w (25% laser power intensity), for a total of 90 s, until ablation of the CF680 signal in the ROI was achieved.

## Quantification And Statistical Analysis

### Two-photon microscopy image analysis

Raw (LSM) image files were imported into the Imaris (Bitplane) software. Movies were stabilised for rotational/translational drift using Imaris correct drift function, using an appropriate fiducial marker as a reference (e.g., stationary macrophage, mouse hair, collagen capsule).

### Cell tracking

Tracking and segmentation of cells (including B cells and macrophages) were performed with Imaris surfaces function, using a resolution of 0.8 μm. Thresholds were chosen in a semi-automated manner on the Kaede (green) channel and tdTomato (red) channel for B cells and macrophages, respectively. Surfaces were then tracked using Imaris tracking function with parameters: Brownian motion, max gap size = 0, max distance = 7 μm, and filtered to retain tracks with duration greater than 150 s (5 time steps) to remove short-lived/noisy tracks.

Tracking of Cyan OT-II T Cells required an additional filtering step because the SHG signal and cyan signal overlap in the blue channel. A surface was drawn at resolution 20 μm and threshold chosen in a semi-automated manner to segment the collagen capsule. A mask was drawn on the capsule surface such that only the blue signal outside of the capsule was copied to a new channel, (“T cell channel”). Tracking and segmentation of T cells was then performed as in the B cells and macrophages on the T cell channel.

### Fragment tracking

Tracking and segmentation of fragments, due to their small size and weak signal, required additional steps to avoid capturing full size cells and noisy signal. This was performed as follows: Surfaces (called “surface 1”) were drawn on the green (Kaede) channel at 0.8μm resolution, with threshold chosen in a semi-automated manner to capture both full-size cells and fragments without excess merging of separate objects. Surface 1 was then filtered by volume to retain objects larger than 100μm^3^. The green (Kaede) channel was then masked by surface 1 such that only the green signal *outside* of surface 1 is copied to a new channel (“Mask Channel”). Surface 2 is then drawn on Mask Channel at 0.8μm resolution, with threshold chosen in a semi-automated manner to capture fragments. These were then tracked using Imaris tracking function with parameters: Brownian motion, max gap size = 0, max distance = 7 μm, and filtered to retain tracks with duration greater than 150 s (5 time points) to remove short-lived/noisy tracks.

### Filament tracing

Tracking and segmentation of macrophage filaments, due to their rapid movement and weak signal compared to the bright macrophage body, was not able to be performed in a fully automated manner. Instead, this was performed in a semi-supervised manner: For each frame, the centroid of the macrophage was identified automatically using Imaris FilamentTracer function. Individual filament tips were then manually identified in each frame. Using the Imaris Edit Filaments tool, filaments were drawn from the macrophage body to the tip in a semi-supervised manner wherein the filament thickness is calculated based on signal intensity. Filament tips were then manually tracked on a frame-by-frame basis, by adding filament tips to their respective tracks. The total track length, for a single TBM, is determined by summing individual filament tip track lengths of each filament originating from that TBM over the course of the imaging session.

### Vacuole counting

Macrophage vacuoles were manually counted on a single z-slice, based on visually inspecting a lack of signal within the macro-phage body.

### Germinal center segmentation and analysis

The GC structure was identified in a semi-supervised manner using Imaris surfaces function on the green (Kaede) channel at resolution 40μm. All surfaces except the largest volume surface (the GC) were then removed from the surface object.

### Imaging statistical tests

All imaging statistics were exported using Imaris export statistics function. Plotting and statistical analysis were performed in R v4.1.2 using bespoke scripts for data wrangling. p-values were calculated using Wilcoxon Rank-Sum test (wilcox.test() function) or Kolmogorov-Smirnov tests (ks.test() function). Plots were exported using ggplot (ggsave function) to.svg image format. Comparison between two groups was performed using unpaired Student’s *t* test in Prism. Sample size and p values are reported in the figure and figure legends.

### Nearest-neighbour analysis

Macrophage surface objects were drawn using Imaris Surfaces function on the Red signal located within a germinal center. Surface XYZ coordinates were exported as.csv and imported into R. Nearest-neighbour distances between objects in each germinal center were calculated in 3D using nndist() function from the spatstat package. Analysis was repeated on three independent images. Monte Carlo sampling was performed by randomly placing non-overlapping spheres of radius 10μm (representing macrophages) within a sphere of radius 81 μm (representing the GC). For each germinal center, sampling was conducted until n = 18 macrophages filled the simulated GC. This was repeated for n_run = 50 GCs for a total of 900 samples. Nearest-neighbour distances between randomly positioned spheres was calculated as above.

### Time-projection

Movies were time cropped to 10 intervals (e.g., frames 1-60, 1-54, 1-48, etc.) for a 60-frame movie. For each crop, MATLAB Time Projection function was used to integrate signal across all time points. Surfaces were drawn using Imaris surfaces function in a semi-supervised manner for the full-length time projected dataset (e.g., frames 1-60). This threshold was then used for the remaining crops (e.g., frames 1-54, 1-48, etc).

### Single-cell RNA-seq data processing

Sequencing reads were mapped to an mm10–3.0.0 reference transcriptome, quantitated using cellranger count, and consolidated into a single gene expression count matrix using cellranger aggr. HTO and ADT count matrices were obtained using CITE-seq-count. Preprocessing and QC was performed in R. Cells were excluded from further analysis if more than 25% of the genes expressed were mitochondrial genes, if there was no ADT and HTO data for the corresponding cell barcode, or if more than one hashtag was detected. 11,384 cells passed library and cell QC. The data was normalised using SCTransform, and the top 3000 variable genes were used as input to NMF for dimension reduction. Ranks 2 to 14 were tested with 50 iterations of the Brunet algorithm of NMF.^33^ The cophenetic coefficient was high at ranks 5,7 and 10. The data was factorised at rank 10 into an *H* matrix, which scores each cell on cluster membership, and a *W* matrix, which describes the contribution of each gene to each cluster. The decomposed dataset was visualised using Similarity Weighted Non-negative Embedding (SWNE).^34^

### Differential gene expression and gene set enrichment analysis (GSEA)

Raw counts were aggregated to the level of HTOs (mouse replicate) and populations identified by NMF. The pseudobulked data was analyzed using edgeR to identify differentially expressed genes. GSEA was performed using the mouse GO geneset (M5.all.v0.3.symbols.gmt) with 1000 permutations of genesets. Enriched pathways with p value <0.05 and FDR q-value <0.15 were visualised as networks using the EnrichmentMap app in Cytoscape. Clustered pathways were identified and summarised with the AutoAnnotate app.

### Agent-based modeling

Agent-based modelling of TBM and fragment dynamics were conducted in the MASON simulation library for Java, ^87^ with a modified version of the code from.^88,89^

The simulation space is defined as a sphere with equivalent volume to the mean GC volume measured through Imaris surface function, and the simulated time step is 15 s. For each simulation, either 18 stationary TBMs or 18 migratory TBMs (mean number of TBMs observed) were simulated as non-overlapping spheres with diameter *d*, placed in the sphere randomly such that their mean nearest neighbor distance corresponded to the observed distances measured in the imaging data. *D* = 27μm was chosen as the representative diameter because this produces a sphere with volume equal to the mean volume of an imaged TBM. We note that the effective coverage of a TBM is higher than its true volume, and therefore our simulated volume is a conservative estimate.

Fragments were simulated as n = 10–7,000 non-overlapping spheres of diameter 3.54 μm (corresponding to the imaging data Figure S1G), initially positioned at random in the simulated volume. Fragments were simulated to move with a random walk defined by calibration of motility parameters to the imaging data (see below). Upon contact with a TBM, the corresponding fragment is removed from the simulation, and the count of removed fragments is increased by one. To keep the fragment density constant during the simulation, each removed fragment is replaced by a new fragment spawned at a random position in the volume. Simulations were run for 75 simulated minutes and fragment count data and position data for calibration were exported as.csv and plotted in ggplot2 in R v4.1.2 using bespoke scripts.

Calibrating the simulated fragment motility to match the imaging data was performed through a broad search of simulated motility parameters. The calibration was performed in three main steps.

### Calibrating simulated fragment mean speed

Observed fragment speed data was exported from Imaris and imported into R. Bespoke R scripts were used to plot the distribution of fragment speed. Fragment speeds were observed to be log normally distributed, and thus were fitted to a lognormal distribution using the fitdistr() function from the *fitdistrplus* package (Figure S3A). Simulated fragment speeds were then sampled from the fitted distribution (Figure S3B):

X∼LogN(μ=1.17,σ=0.335)

### Calibrating simulated fragment turn speed

Observed position data was exported from Imaris and imported into R. Bespoke R scripts were used to calculate the turn angles for every time step for every track. Fragment turn angles were fitted to a Beta distribution using the fitdistr() function from the *fitdistrplus* package (Figure S3C). Simulated fragment turn speeds (pitch and roll) were then sampled from the fitted distribution:

X∼Beta(α=2.02,β=1.707)

### Calibrating simulated fragment meandering index

Observed fragment speed data was exported from Imaris and imported into R. Bespoke R scripts were used to plot the distribution of fragment speed. Fragment meandering indices were observed to be log normally distributed, and thus were fitted to a lognormal distribution using the fitdistr() function from the *fitdistrplus* package (Figure S3D). Meandering index (MI) is an emergent property of the simulation, rather than a parameter, and therefore the simulated MI was not able to be directly sampled from the lognormal distribution. Instead, we introduced a ‘meanderChance’ parameter for each cell. This parameter corresponds to the probability of a fragment performing a ‘meander’ in each time step. A ‘meander’ is defined as a non-random movement directly away from the track start position, for a single simulated timestep. This parameter was drawn from a lognormal distribution for each cell. For this distribution, we varied and and empirically compared the differences in MI between the simulated and the observed for each parameter set. Observed motility data was exported from Imaris and imported into R. Bespoke scripts were used to plot observed MI and the MI of simulated fragments for each parameter set. To determine if the simulated MI was true to the observed, we plotted empirical cumulative distribution functions of both simulated and observed datasets (Figure S3E). We used two-sided Anderson Darling test from *kSamples* package in R to test the differences between the two distributions for each parameter set. We found that parameter values and resulted in a null hypothesis (p = 0.561) between the simulated and observed MIs. This parameter set was used for all fragment simulations.

## Additional Resources

### Animation

Time-lapse images were storyboarded using Imaris animation tool and exported, compiled and annotated in AfterEffects (Adobe), and exported to.mp4 using Adobe Media Encoder.

## Supplementary Material

Table S1

Table S2

Table S3

Table S4

Video S1

Video S2

Video S3

Video S4

Video S5

Video S6

Supplemental figures

## Data Availability

Single-cell RNA-seq data have been deposited at GEO and are publicly available as of the date of publication. Accession numbers are listed in the key resources table. Intravital imaging, confocal microscopy and FACS data reported in this paper will be shared by the lead contact upon request. All original code has been deposited at Zenodo and is publicly available as of the date of publication. DOIs are listed in the key resources table. Any additional information required to reanalyze the data reported in this paper is available from the lead contact upon request.

## Highlights

1. TBMs are lymph node-resident, CD169-lineage, CSF1R-blockade-resistant macrophages
2. TBM precursors in the follicle are activated by local cell death
3. TBMs are stationary and uniformly dispersed throughout the germinal center
4. TBMs use cellular processes to search for and capture motile apoptotic cell fragments
