# Supplementary material for: Apoptotic cell fragments locally activate tingible body macrophages in the germinal center
Source: Cell. Author manuscript; Available in PMC 2023 May 5. (PMC7614509; doi:10.1016/j.cell.2023.02.004)
Supplement: Supplemental figures [file EMS174789-supplement-Supplemental_figures.pdf]

Supplemental figures

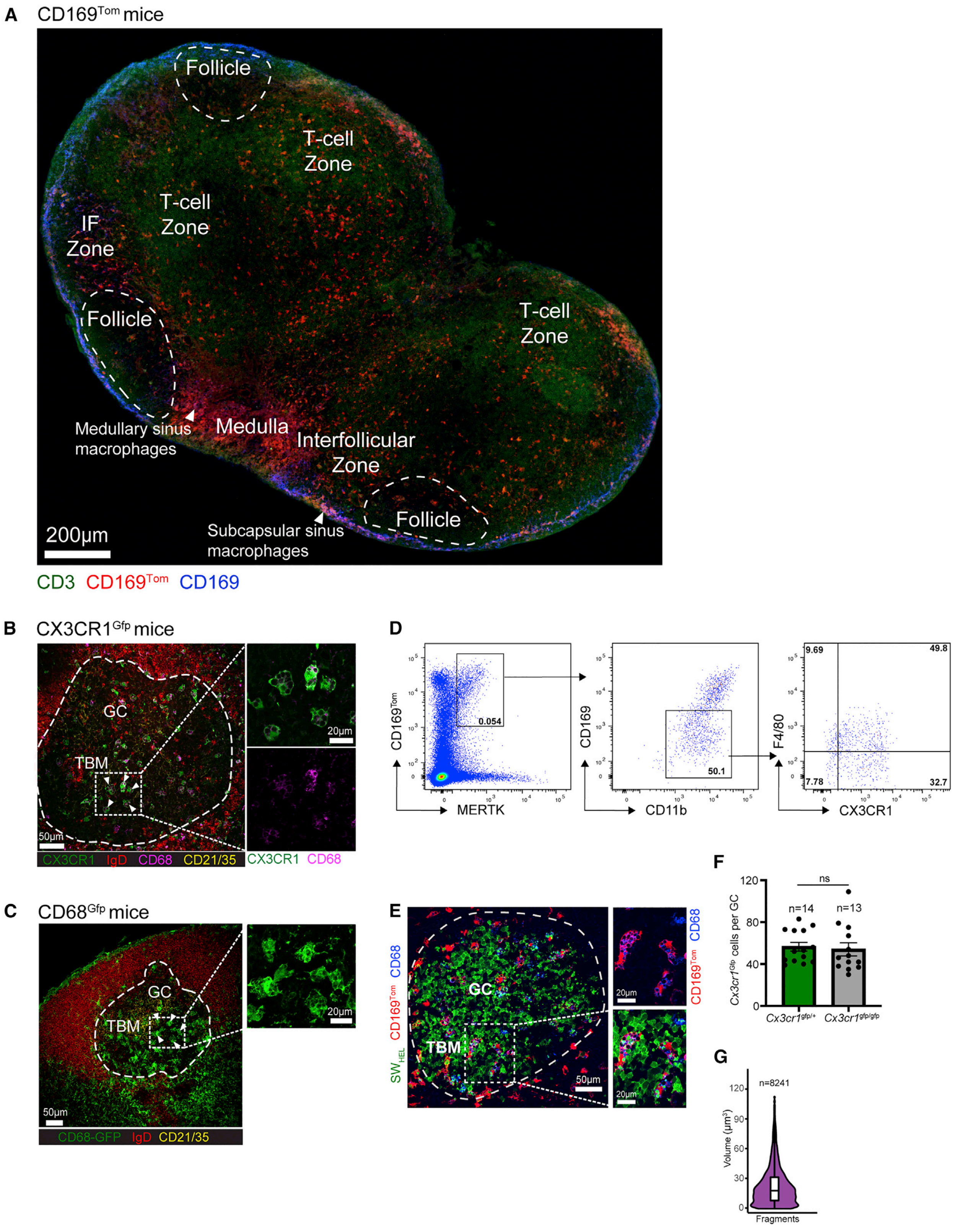

(legend on next page)

Figure S1. Reporter systems for tracking TBMs *in vivo*, related to Figure 1

(A) Mosaic-tiled image of a representative lymph node section from a $CD169^{Tom}$ mouse stained with CD3 (green) and CD169 (blue). IF, interfollicular zone.

(B) GFP expression by GC TBMs in $CX3CR1^{Gfp}$ mice co-stained with IgD (red), CD68 (antibody, magenta), and CD21/35 (yellow). Confocal analysis of inguinal lymph nodes on day 7 post CGG/AddaSO3 s.c. immunization.

(C) GFP expression by GC TBMs in $CD68^{Gfp}$ mice co-stained with IgD (red) and CD21/35 (yellow). Confocal analysis of inguinal lymph nodes on day 7 post CGG/AddaSO3 s.c. immunization.

(D) FACS analysis of $CD169^{Tom}$ mice for expression of MERTK, CD169, CD11b, F4/80, and CX3CR1.

(E) Confocal analysis of a $CD169^{Tom}$ lymph node on day 9 post immunization with HEL-OVA showing TOM+ cells inside GC contain $CD68^{+}$ vacuoles.

(F) Numbers of GFP + TBMs in GCs from $CX3CR1^{Gfp/wt}$ (heterozygous reporter) and $CX3CR1^{Gfp/Gfp}$ (homozygous knock-out) mice. Quantitation from immunofluorescence microscopy of inguinal lymph nodes. Data from one experiment, three mice per group.

(G) Volume of fragments from intravital microscopy and image analysis using the surfaces feature in Imaris.

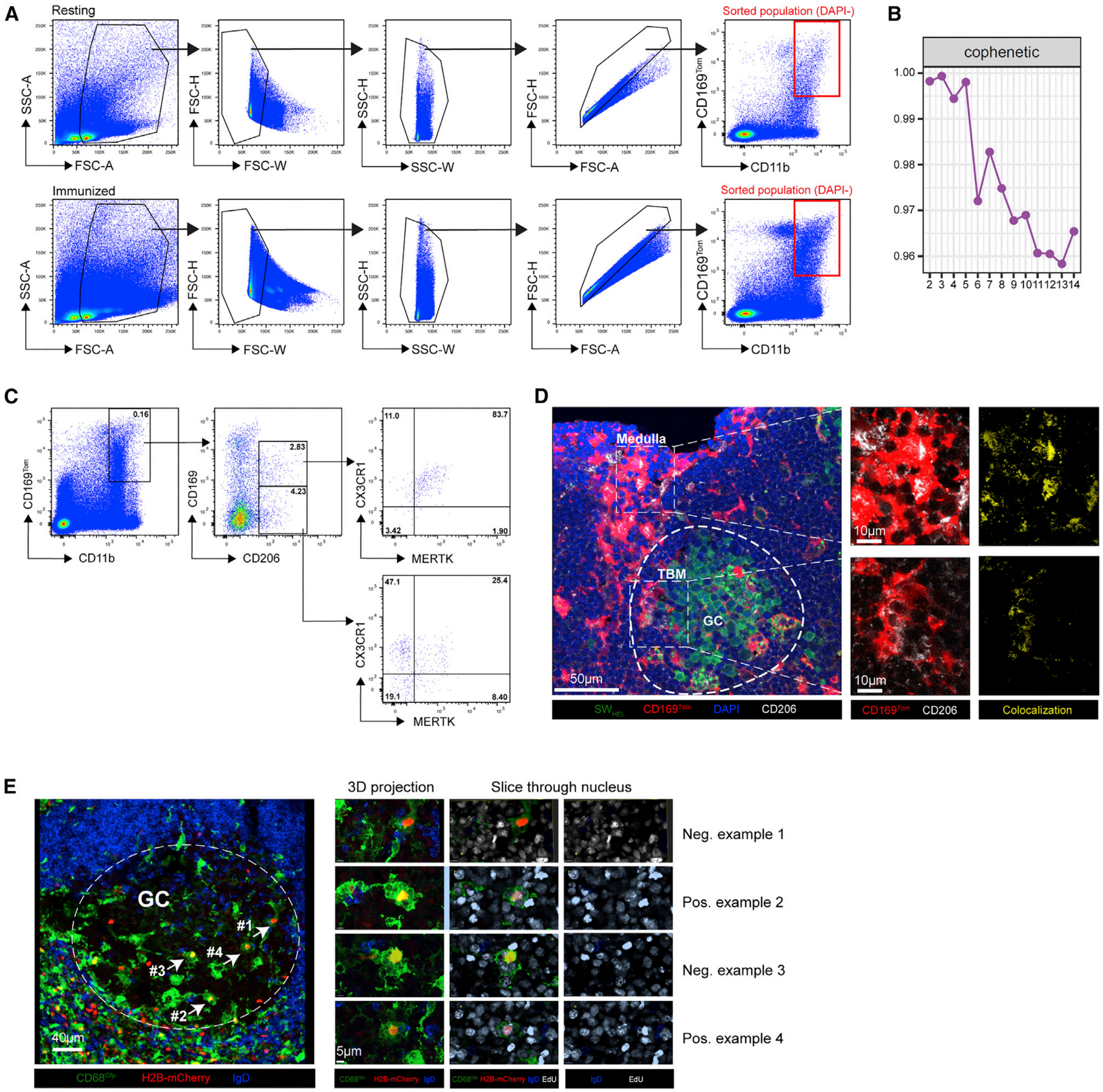

Figure S2. Isolation of CD169-lineage cells for CITE-seq, related to Figure 4

(A) FACS sorting gates for CITE-seq experiment. CD169^{Tom}CD11b^{+} DAPI^{-} cells were sorted (red gate) from both resting and immunized mice.

(B) Cophenetic correlation coefficient scores from 50 iterations of NMF at ranks 2 through to 14.

(C) FACS analysis of day 12 immunized lymph nodes from CD169^{Tom} mice for CD206 and CX3CR1 expression on TOM^{+}CD11b^{+}CD169l^{ow} and TOM^{+}CD11b^{+}CD169^{-}cells.

(D) Confocal microscopy showing expression of CD206 in TOM+ cells in medulla and GC of CD169^{Tom} mice.

(E) Confocal images of a GC and zoomed TBMs, where TBM nuclei were marked as H2B-mCherry+ in CX3CR1-CreERT2, Rosa_LSL_H2b-mCherry, CD68-Gfp mice. EdU treatments were provided continuously from day 1 post immunization with GCC/AddSO3, with harvest on day 11.

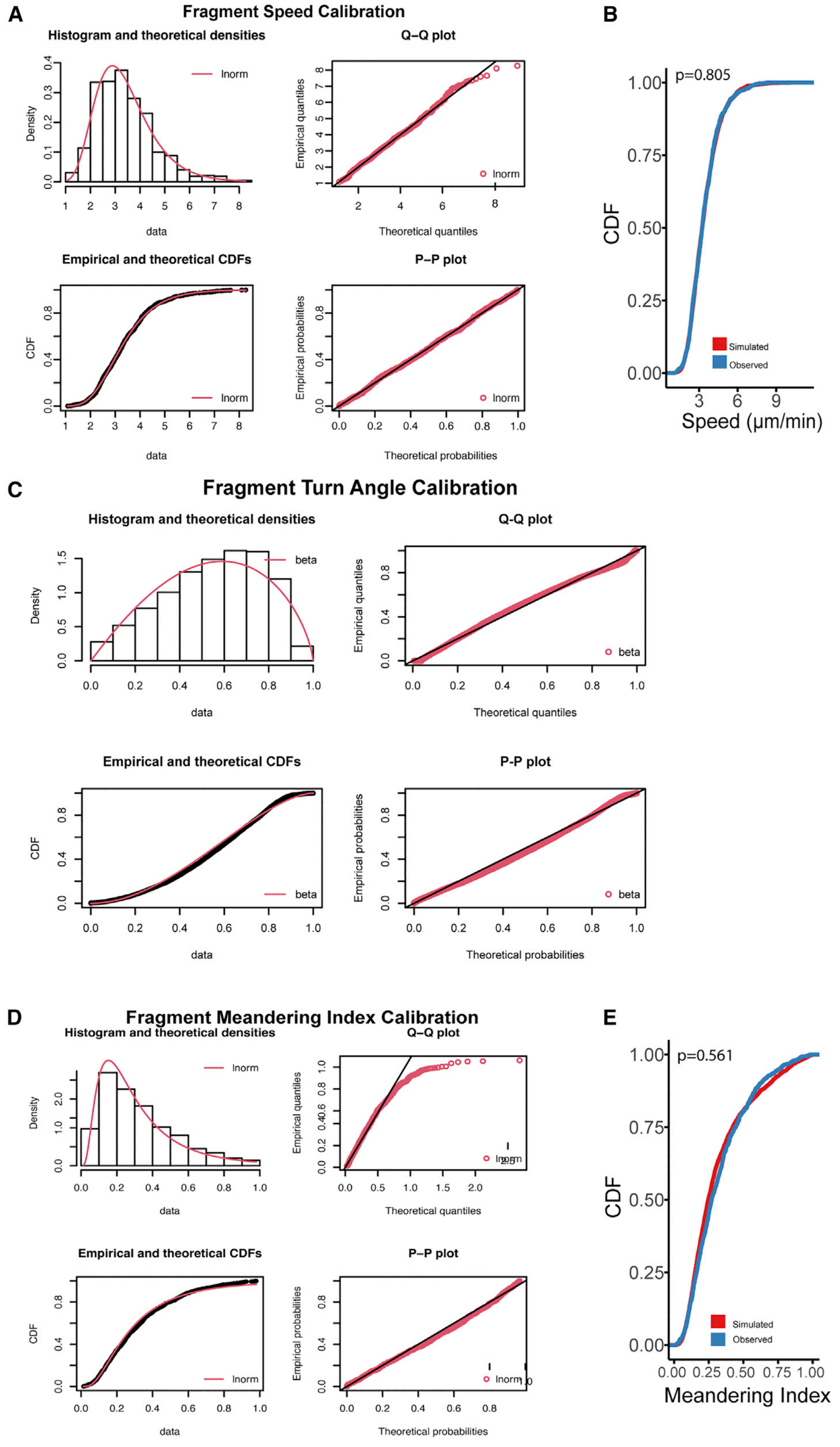

(legend on next page)

Figure S3. Mathematical modeling of TBM and apoptotic cell fragment dynamics, related to Figure 3

(A) Histogram (top-left), Q-Q plot (top-right), CDF plot (bottom-left), and P-P plot (bottom-right) of observed fragment speeds compared to lognormal fit (red, mu = 1.17, delta = 0.335).

(B) CDF plot of observed fragment speeds compared to simulated fragment speeds.

(C) Histogram (top-left), Q-Q plot (top-right), CDF plot (bottom-left), and P-P plot (bottom-right) of observed turn angles compared to a beta fit (red, alpha = 2.02, beta = 1.707).

(D) Histogram (top-left), Q-Q plot (top-right), CDF plot (bottom-left), and P-P plot (bottom-right) of observed meandering index (MI) compared to a lognormal fit (red, mu = -3, delta = 1.5).

(E) CDF plot of observed fragment MI compared to simulated MI.

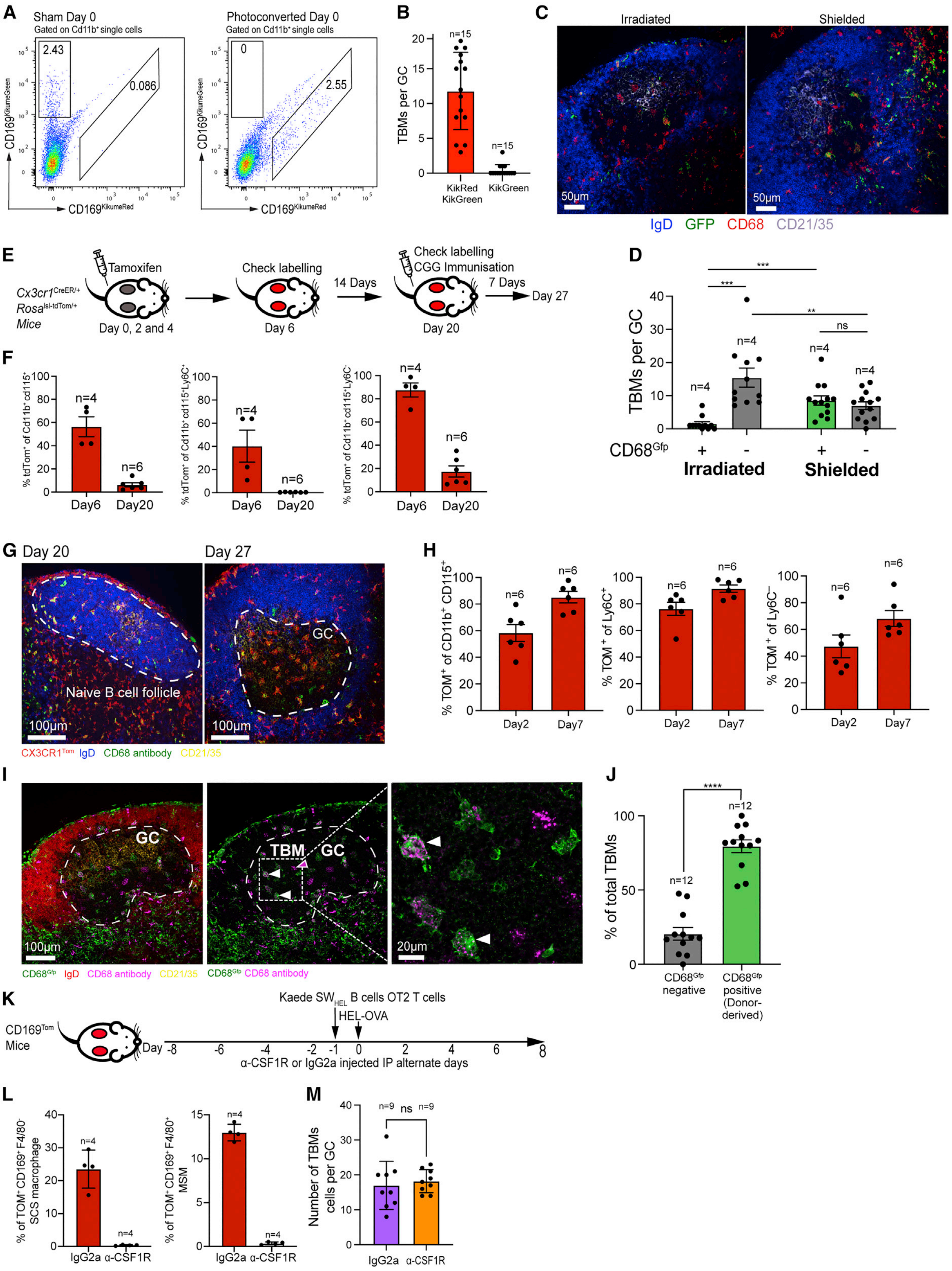

(legend on next page)

Figure S4. Origin of TBMs, related to Figure 5

(A) FACS plot of sham and photoconverted CD169^{Kik} inguinal resting lymph nodes showing unphotoconverted KikumeGreen vs. photoconverted KikumeRed for CD11b^{+} single cells.

(B) Quantification of the number of TBMs per GC that were photoconverted or not photoconverted at day 7 post-HEL-OVA immunization. n = number of GCs; data are representative of 3 independent experiments.

Whole-body irradiation experiment

(C) Wildtype non-fluorescent mice were lethally irradiated and reconstituted with CD68^{Gfp} bone marrow. Mice were immunized s.c. with CGG/AddaSO3 >8 weeks later. Confocal image of a GC, showing follicular mantle (IgD, blue), follicular dendritic cell network (CD21/35, gray), macrophages (CD68 antibody, red), and donor-derived GFP (green).

(D) Quantitation of (C) the proportion of CD68^{+} (antibody) GC TBMs that were CD68-GFP+. Data points are individual GCs from 11 mice and 2 independent experiments. Mean (+/− S.E.M.).

Stable labeling of tissue-resident macrophages

(E) Schematic for fate mapping the contribution to GC TBMs of cells that were stably expressing CX3CR1 prior to immunization. Immunization, CGG/AddaSO3.

(F) Quantification of blood monocytes and inflammatory blood monocytes (Ly6C+) at the indicated time points post-tamoxifen.

(G) Confocal image of a non-immunized naive follicle (Day 20) and post-immunization GC (Day 27), showing follicular mantle (IgD, blue), follicular dendritic cell network (CD21/35, mustard), macrophages (CD68 antibody, green), and tdTomato (red).

Contribution of circulating monocytes

(H) Related to Figure 5E. Quantification of blood monocytes and inflammatory blood monocytes (Ly6C+) at the indicated time-points post-tamoxifen.

Shielded irradiation experiment

(I) CD68^{Gfp} mice received lethal whole-body X-ray irradiation. Individual inguinal lymph nodes from opposite body sides were left exposed (non-shielded) or lead shielded. Mice were subsequently reconstituted with WT non-fluorescent bone marrow. 8–10 weeks later, mice were immunized s.c. with GCC/AddaS03, with analysis on day 8. GC TBMs were identified by antibody staining for CD68 (magenta), IgD (red, follicular mantle), and CD21/35 (mustard, FDCs). GFP expression by TBMs was assessed.

(J) Quantitation of the frequencies of CD68^{+} (antibody) GC TBMs that were GFP+ in non-shielded or shielded lymph nodes. Data points are individual GCs, pooled from 5 mice and 2 independent experiments. INSERT NEW LINE

CSF1R blockade

(K) Schematic for anti-CSF1R experiment to deplete resident SSMs and MSMs prior to and during HEL-OVA immunization.

(L) Quantification of CD169^{Tom}CD11b^{+}CD169^{+}F4/80^{-} SSM (left) and CD169^{Tom}CD11b^{+}CD169^{+}F4/80^{+} MSMs (right) from mice treated with IgG2a or anti-CSF1R m-Ab. n = number of individual mice.

(M) Quantification of number of TBMs per GC in day 8 response lymph nodes of CD169^{Tom} mice treated with IgG2a isotype control antibody (left) or anti-CSF1R blocking antibody (right). n = number of GCs; data are representative of 3 independent experiments.
